# NDR1/FBXO11 promotes phosphorylation-mediated ubiquitination of β-catenin to suppress metastasis in prostate cancer

**DOI:** 10.7150/ijbs.98907

**Published:** 2024-09-16

**Authors:** Zuodong Xuan, Chen Chen, Huimin Sun, Kunao Yang, Jinxin Li, Meilin Fu, Yang Bai, Zeyuan Zheng, Yue Zhao, Chunlan Xu, Bin Liu, Tian Li, Chen Shao

**Affiliations:** 1 Department of urology, Xiang'an Hospital, Xiamen University, Xiamen, China.; 2 Department of urology, First People's Hospital of Linping District, Hangzhou, China.; 3 School Of Medicine, Xiamen University, Xiamen, China.; 4 School of Basic Medicine, Fourth Military Medical University, Xi'an 710032, China.

**Keywords:** Phosphorylation, Ubiquitination, Epithelial-mesenchymal transition, Prostate cancer, Metastasis, FBXO11, NDR1

## Abstract

**Background:** Prostate cancer progression hinges on β-catenin's stability and activity, a key factor in epithelial-mesenchymal transition (EMT) and metastasis. This study delves into NDR1-dependent phosphorylation's impact on β-catenin via FBXO11, an E3 ubiquitin ligase, in prostate cancer cells.

**Methods:** Human prostate cancer cell lines underwent various *in vitro* assays, including real-time PCR, Western blotting, immunoprecipitation, immunofluorescence, and protein stability assays, to explore β-catenin's interactions and post-translational modifications. NDR1 modulation's *in vivo* efficacy was assessed using a nude mice lung metastasis model. Small-molecule screening identified a potential NDR1 activator, aNDR1, tested for its effects on metastasis via *in vitro* and *in vivo* assays.

**Results:** NDR1 phosphorylated β-catenin at Ser33/37, facilitating its interaction with FBXO11. This led to FBXO11-mediated ubiquitination and cytoplasmic degradation of β-catenin, while the NDR1-FBXO11 complex impeded β-catenin nuclear translocation by inducing JNK2 ubiquitination. Thus, NDR1 and FBXO11 jointly regulate β-catenin activity in prostate cancer cells through dual phosphorylation-driven ubiquitination, potentially suppressing EMT. Reduced NDR1 expression inhibited FBXO11 and β-catenin phosphorylation, diminishing β-catenin and JNK2 ubiquitination, promoting EMT and enhancing prostate cancer cell metastasis. The inhibitory effects of aNDR1 on prostate cancer metastasis were validated.

**Conclusion:** The NDR1/FBXO11 axis outlines a non-canonical β-catenin degradation pathway crucial in regulating EMT and prostate cancer cell metastasis. NDR1 activation, particularly with aNDR1, could offer a promising therapeutic avenue against prostate cancer metastasis.

## 1. Introduction

Neoplasms remain the main killer worldwide 1-4. Among which, prostate cancer was the most prevalent malignancy among males in 2023, comprising 29% of cases, and ranked second in male cancer-related mortality 5, 6. Metastasis is the primary cause of treatment failure, during which cells undergo epithelial-mesenchymal transition (EMT), characterized by the transformation of epithelial cells into mesenchymal cells, accompanied by alterations in cell morphology, reduced cell adhesion, and enhanced cell motility and invasion 7-9. Importantly, the EMT process is pivotal in facilitating the invasion and metastasis of prostate cancer cells 10, 11.

Various signaling pathways and regulatory factors, including Wnt/β-catenin, PI3K/Akt, and TGF-β/Smad, have been shown to promote EMT, resulting in cells acquiring mesenchymal characteristics and increased migratory capacity 12-18. Among these, β-catenin is essential for cell adhesion and regulates both cell-cell adhesion and signal transduction. Under normal conditions, cellular levels of β-catenin are tightly regulated to maintain normal cellular functions 19. β-catenin degradation primarily occurs through ubiquitination and subsequent proteasomal degradation, facilitated by a protein complex comprising Axin, Adenomatous Polyposis Coli (APC), Glycogen Synthase Kinase-3β (GSK-3β), and Casein Kinase 1 (CK1), which prevents β-catenin accumulation within the cell 20, 21. However, dysregulation of β-catenin degradation can lead to abnormal cell proliferation and transcription activation and may even contribute to tumorigenesis and tumor progression 20. β-catenin plays an oncogenic role in various malignancies. In diffuse large B-cell lymphoma (DLBCL), exosomal ENO2 enhances glycolytic metabolism, activating the GSK3β/β-catenin/c-Myc signaling pathway, which in turn promotes macrophage M2 polarization 22. In triple-negative breast cancer (TNBC), β-catenin stability and function are restricted by LYPLAL1-DT, thereby inhibiting malignant progression and EMT 23. Consequently, therapeutic strategies targeting β-catenin must be tailored to the specific cancer type and the underlying mechanisms involved.

The HIPPO signaling pathway is a highly conserved cascade involved in regulating cell proliferation, cell fate determination, and tissue development, among other biological processes 24. Studies have indicated that members of the HIPPO pathway, such as YAP and TAZ, along with their downstream effectors LATS1/2 and MOB1A/1B, can influence the activity and transcriptional regulatory capacity of β-catenin 25-28. NDR1, a crucial kinase within the HIPPO pathway, plays a role in regulating cell proliferation, apoptosis, and tissue morphology maintenance 29, 30. However, the impact of NDR1 on prostate cancer cells has been rarely reported, and the mechanisms underlying β-catenin dysregulation in prostate cancer remain poorly understood. Further investigations are urgently needed to elucidate the factors triggering β-catenin dysregulation, the complex regulatory mechanisms governing β-catenin stability and activity, and the role of NDR1 in these processes.

Herein, this study hypothesizes that NDR1 kinase activity plays a significant role in prostate cancer, specifically in influencing the epithelial-mesenchymal transition (EMT) and the regulatory mechanisms governing β-catenin degradation. The results demonstrate the inhibitory effect of NDR1 kinase activity on the EMT of prostate cancer and reveal a non-canonical pathway of β-catenin degradation. This pathway relies on NDR1-mediated phosphorylation of the E3 ubiquitin ligase FBXO11 at Ser187 and β-catenin at Ser33/37. Additionally, the study elucidates the signaling pathway involved in the NDR1-FBXO11-mediated ubiquitination of JNK2, which regulates the nuclear translocation of β-catenin. These findings shed light on a regulatory mechanism governing the stability and activity of β-catenin in prostate cancer cells. Expanding upon these discoveries, the potential clinical application of these pathways was explored by screening and synthesizing small-molecule activators targeting NDR1, assessing their ability to activate the NDR1 pathway, and evaluating their inhibitory activity on the EMT in prostate cancer and metastasis. Overall, this research provides valuable insights into the mechanisms underlying β-catenin dysregulation, EMT and metastasis in prostate cancer, contributing to a deeper understanding of prostate cancer development, potentially enabling risk prediction, and facilitating the exploration of novel therapeutic strategies.

## 2. Material and methods

### 2.1 Cell lines and cell culture

The currently common metastatic androgen receptor-independent prostate cancer cell lines in the market are PC3 and DU145 31-33. Human prostate cancer cell lines (LNCaP [Cat. No.CRL-1740], C4-2 [Cat. No. CRL-3314], PC3 [Cat. No. CRL-1435], DU145 [Cat. No. HTB-81]) and human renal epithelial cells (293T) were purchased from the American Type Culture Collection (ATCC, Manassas, VA, USA). PC3 and DU145 cell lines were cultured in 1640 medium (Cat. 11875093, Gibco, Waltham, MA, USA) supplemented with 10% fetal bovine serum, while 293T cells (Cat. No. CRL-3216) were cultured in DMEM medium (Cat. No. 11965092, Gibco) supplemented with 10% fetal bovine serum. All cell lines were supplemented with 100 U/ml penicillin and 100 µg/ml streptomycin and maintained at 37°C in a humidified atmosphere with 5% CO_2_.

### 2.2 Cell transfection

Transfection of plasmid DNA, siRNA, and shRNA was performed using Lipofectamine 3000 (ThermoFisher, USA) for single or co-transfection. The following constructs were used in this study: Myc-NDR1, Flag-FBXO11, His-beta-catenin, Myc-PMCV (Sino Biological), and HA-Ub (Addgene, MA, USA). The shRNA vectors used were: FBXO11 shRNA Vector, NC shRNA Vector, GAPDH shRNA Vector, and β-TrCP shRNA Vector (Gene Pharma, Shanghai, China).

### 2.3 Reverse transcription polymerase chain reaction (RT-PCR)

Total RNA was extracted from the cells using TRIzol RNA Isolation Reagents (ThermoFisher Scientific, Waltham, MA, USA), following the manufacturer's instructions. For the miRNA reverse transcription, the All-in-One™ miRNA First-Strand cDNA Synthesis Kit (GeneCopoeia, Rockville, MD, USA) was used, and for reverse transcription of the general genes, the PrimeScript™ RT Master Mix (Clontech Laboratories, CA, USA) was employed. Real-time PCR was conducted using SYBRGreen PCR Master Mix (Applied TaKaRa, Otsu, Shiga, Japan) on the CFX96 Deep Well Real-time PCR Detection System (BioRad, Hercules, CA, USA). The primer sequences for all specified genes are listed in Supplementary Table 1.

### 2.4 EMT stimulation

Recombinant human TGF-β1 (R&D Systems, Minneapolis, USA; 240-B) was utilized at a concentration of 10 ng/ml to induce EMT in prostate cancer cells, as TGF-β is a known potent inducer of EMT 34, 35. The cells were evenly seeded in culture dishes at a density of 0.9-1.0 x 10^4^ cells/cm^2^. Subsequently, the culture medium containing 1X EMT inducer TGF-β (10 ng/ml; R&D Systems) was added, and the cells were then incubated in a 37°C/5% CO_2_ incubator. Cell morphology was monitored daily. After three days of seeding, the culture medium was replaced with fresh preheated medium supplemented with 1X EMT induction medium supplement, and the cells were further incubated in a 37°C/5% CO_2_ incubator. After five days of seeding, the cells were prepared for analysis and cell morphology was observed using an inverted microscope.

### 2.5 Scratch, migration, and invasion assays

For the scratch assay, cells from both experimental and control groups were digested and quantified into single-cell suspensions. The cells were evenly distributed into 6-well cell culture plates, with each well containing 1 × 10^6^ cells in 2 ml of culture medium. Before the scratch assay, the cells were treated with mitomycin-C (Yeasen, Shanghai, China, CAS 50-07-7) (1 μg/ml) for 1h to inhibit cell proliferation. After 24h of incubation in a CO_2_ incubator, a scratch was created in the designated area using a sterile 200 μl pipette tip. The wells were subsequently washed with PBS, and photographs were captured at 0h as a reference point. Fresh culture medium was then replenished, and the cells were further incubated. Photographs were taken at regular intervals to monitor the scratch area and analyzed using the Image J software (National Institutes of Health, Bethesda, MD, USA).

For the cell Transwell assay, well-growing cells from both experimental and control groups were serum-starved for 8 hours. Afterward, the cells were digested and counted, and 1 × 10^4^ cells were added to the upper chamber of a Transwell 24-well plate (Corning, Corning, NY, USA). The upper chamber contained 200 μl of cell culture medium with 0.2% BSA (Goldbio, St Louis, MO, USA), while the lower chamber contained 500 μl of cell culture medium with 20% FBS (Goldbio)for chemoattraction. After 24 hours of incubation in a CO_2_ incubator, the upper and lower chamber culture media were removed. The cells on the lower chamber were fixed with 4% PFA (Aladdin, Shanghai, China), washed with PBS, and stained with crystal violet for 20 minutes. Subsequently, the cells attached to the lower chamber were retained, and photographs were captured under a 200× magnification microscope. Finally, the cell numbers were counted using the Image J software (National Institutes of Health).

For the assessment of cell invasion ability, the upper chamber side of the Transwell membrane (Corning) was coated with a matrix gel. The subsequent steps were the same as those for the cell migration assay. The matrix gel was prepared by mixing it with serum-free culture medium at a ratio of 1:8. Once 50 μl of the diluted matrix gel was added to the upper chamber and evenly spread, the plate was incubated overnight in a CO_2_ incubator before use.

### 2.6 Protein stability assay

The control and experimental cell groups were evenly cultured in 5 sets of 60mm cell culture dishes until reaching approximately 70% confluence. Then, 100 μg/ml of cycloheximide (CHX, Sigma) was added to the cells in the first group to inhibit protein synthesis, and the time point was marked as 24 hours. The cells were further cultured, and at intervals of 4 hours, an equivalent amount of CHX was added to the first group, marking the time points as 12 hours, 8 hours, and 4 hours, respectively, until the final time point was marked as 0 hours. Cell proteins were collected, and Western Blot analysis was conducted to assess the changes in β-catenin protein expression levels. The extent of protein degradation and stability were determined based on the intensity of the observed bands.

### 2.7 Prokaryotic protein expression

The pET-GST/β-cateninS1, pET-GST/β-cateninS2, pET-GST/β-cateninS3 and pET-GST/NDR1 expression plasmids, purchased from Sino Biological, were transformed into Escherichia coli DH5α. Single colonies were selected and cultured overnight in an LB liquid medium containing kanamycin at 37°C. Plasmids were extracted, and their identity was confirmed through restriction enzyme digestion and DNA sequencing. Afterward, the correct recombinant plasmids were transformed into BL21(DE3) cells (Beyotime, D1013S). Positive clones were selected on LB agar plates containing kanamycin (Beyotime) and then inoculated into an LB liquid medium supplemented with kanamycin. When the OD value reached 0.6-0.8, 0.5 mmol/L IPTG (Beyotime) was added, and the cells were cultured at 37°C with shaking. Samples were collected at 0h, 2h, 4h, 6h, and 8h after IPTG induction, and the supernatant and precipitate were separated by centrifugation. SDS-PAGE was performed to analyze the expression of the fusion protein and determine the optimal conditions for exogenous protein induction. Positive single clones of transformed BL21(DE3) cells were then subjected to large-scale culture. After the OD value reached 0.6-0.8, IPTG was induced for 6h. The cells were harvested, and bacteria were lysed with 8 mol/L urea to remove insoluble fragments. The protein was purified using Ni-NTA resin (Jin Sirui, Nanjing, China) and then dialyzed for refolding. Finally, the purified protein was stored at -80°C.

The primer sequences are provided in the table Supplementary Table 1.

### 2.8 Protein *in vitro* kinase assay

NDR1, an S/T kinase, was artificially activated to its active form using Kinase Buffer (Cell Signaling Technology) and IP experiments in HEK293T cells. In a 0.5 mL ice-cold kinase buffer (1x PBS, 20% glycerol, 4 mM MgCl_2_, 10 mM DTT, protease inhibitor), 0.5 μg of inactive protein (GST-β-catenin expressed in prokaryotes) was added. The mixture was then combined with immunoprecipitated protein, and 100 μM ATP (Sigma-Aldrich, catalog number: A2383-1G) was added. After vortexing, the solution was incubated at 30°C for 15-30 minutes. The reaction was stopped by heating the sample at 100°C for 5 minutes in 2.5x SDS loading dye. Then, SDS-PAGE was performed to separate and detect the protein expression levels of β-catenin.

### 2.9 Site-directed mutagenesis plasmid construction

The wild-type template DNA underwent PCR (BioRad) using high-fidelity pyrobest DNA polymerase (TaKaRa). Subsequently, the PCR product was treated with 1/10 volume of sodium acetate and 1 volume of isopropanol in an ice bath for 5 minutes. After centrifugation, the supernatant was discarded, and a DpnI enzyme (ThermoFisher Scientific) was used for digestion at 65°C for 15 minutes to stop the reaction. The digested product was then transformed into Escherichia coli DH5a strain (Takara, Japan), and mutant clones were selected using antibiotics. Lastly, sequencing was conducted for verification.

Site-directed mutagenesis primer sequences are provided in Supplementary Table 1.

### 2.10 Liquid chromatography-tandem mass spectrometry (LC-MS/MS)

After the enzymatic digestion of protein gel strips, peptide fragments were extracted and analyzed using a tandem mass spectrometer timsTOF pro (Bruker, Manning, MA, USA). Then, the Mascot v2.3.02 software (Matrix Science, Boston, MA, USA) was utilized for database searching. LC-MS/MS analysis was performed at the Analysis and Testing Center of Xiamen University's School of Life Sciences (Xiamen, Fujian, China).

### 2.11 Immunoprecipitation (IP)

The protein lysate was incubated overnight at 4°C with the diluted antibody solution. Protein A/G magnetic beads (Thermo Scientific, USA) were washed with Beads Wash Buffer, and this process was repeated thrice. The washed beads were then stored at 4°C for later use. After balancing, 40 μl of the magnetic beads were added to the IP lysate and incubated for an additional 2-4 hours. The magnetic beads were collected and washed three times with Beads Wash Buffer. Subsequently, the Beads Wash Buffer was discarded, and 40 μl of Loading Buffer (2×) was added to the magnetic beads. The samples were heated at 100°C for 10 minutes and subjected to Western blot analysis.

### 2.12 Immunofluorescence

Cells cultured on glass coverslips were fixed with 4% paraformaldehyde at room temperature for 10 minutes. After rinsing twice with PBS, a blocking buffer (DakoCytomation) was applied and allowed to incubate for 30 minutes. Following this, primary antibodies and fluorescent secondary antibodies were utilized for staining. The primary antibodies used included E-cadherin (Cell Signaling Technology, 3195T, diluted 1:200), β-catenin (Cell Signaling Technology, 8480T, diluted 1:100), β-actin (Cell Signaling Technology, 4970S, diluted 1:300), and NDR1 (Santa Cruz Biotechnology, sc-365555, diluted 1:100). For secondary labeling, Anti-mouse IgG (Alexa Fluor, Irving, TX, USA; #594 Conjugate) (Cell Signaling, 8890, diluted 1:2000) and Anti-rabbit IgG (Alexa Fluor #488 Conjugate) (Cell Signaling, 4412, diluted 1:2000) were used.

### 2.13 Animal model

Six-week-old male BALB/c-nude mice without a thymus were purchased from Xiamen University Experimental Animal Center (Xiamen, China) and were housed in a specific-pathogen-free environment. For the tumor metastasis assay, 2×10^6^ PC3 cells were injected into the tail vein of the mice. Subsequently, every other day, the mice received subcutaneous injections of a small-molecule drug at a concentration of 5 μM, with 0.1 ml administered per mouse, over a continuous 2-week period. The concentration was determined based on our previously published research 36, which demonstrated that this small molecule at such concentration significantly inhibited the viability of prostate cancer cells without affecting the viability of normal prostate cells. The control group received an equal volume of PBS via injection. The mice were euthanized at 40 and 60 days post-injection, and their lungs were examined for metastatic lesions. The animal study was reviewed and approved by The Laboratory Animal Center of Xiamen University (Ethics No.XMULAC20200039). All animal experiments were performed in accordance with the Declaration of Helsinki and "Guide for The Care and Use of Laboratory Animals" 8th Edition.

### 2.14 Immunohistochemistry

Paraffin-embedded tissue blocks were sectioned into 2.5-μm slices and mounted onto glass slides. To inhibit endogenous peroxidase activity, the sections were treated with 3% hydrogen peroxide and then incubated overnight at 4°C with the primary antibody (Cell Signaling Technology, cat: 8480T, dilution: 1:200). Following this, a horseradish peroxidase-conjugated secondary antibody (DakoCytomation, cat: 31460, dilution: 1:5000; Glostrup, Denmark) was applied and left to incubate at room temperature for 1 hour. Visualization of β-catenin expression was achieved through DAB staining (ThermoFisher Scientific, USA), followed by counterstaining with hematoxylin. The primary antibody utilized was β-catenin (Cell Signaling Technology, 8480T, diluted 1:200).

### 2.15 Preparation of phosphorylation antibody

The FBXO11 S187 phosphorylation peptide (synthesized and provided by Xiamen University Antibody Platform, China) was used as an antigen for immunizing rabbits. Initially, 100 μg of the antigen was emulsified with Freund's complete adjuvant (MedChem Express, Texas, USA) and administered subcutaneously at multiple points on the back. Next, three booster immunizations were conducted two weeks apart, with 50 μg of phosphorylation peptide emulsified with Freund's incomplete adjuvant and injected subcutaneously at multiple points on the back. One week after the final immunization, blood samples were collected and allowed to stand at 37°C for 1 hour. Serum was obtained by centrifugation (centrifuge, Beckman, Indianapolis, USA) at 4°C and 12,000 rpm, followed by the addition of an equal volume of glycerol, and then stored at -20°C for subsequent use.

### 2.16 Protein extraction and western blotting

Cell lysates were prepared in RIPA buffer (KeyGEN BioTECH, Jiangsu, China) and quantified using the Bradford protein assay (KeyGEN BioTECH). Nuclear protein extraction was performed using the CelLytic™ NuCLEAR™ Extraction Kit (Sigma-Aldrich, Saint Louis, MO, USA), while membrane and cytoplasmic protein extraction were conducted using the Membrane and Cytosol Protein Extraction Kit (Triton X-114 method) (KeyGEN BioTECH). Subsequently, the lysates were subjected to SDS-PAGE and transferred onto PVDF membranes (Millipore, Belmont, MA, USA). The membranes were then incubated with primary antibodies overnight at 4°C. The following primary antibodies were used: NDR1 (Santa Cruz Biotechnology, Santa Cruz, CA, USA; sc-365555, 1:1000 dilution), β-catenin (Cell Signaling Technology, Danvers, Massachusetts, USA; 8480T, 1:1000 dilution), E-cadherin (Cell Signaling Technology, 3195T, 1:1000 dilution), N-cadherin (Cell Signaling Technology, 13116T, 1:1000 dilution), Claudin1 (Cell Signaling Technology, 13255T, 1:1000 dilution), ZO-1 (Cell Signaling Technology, 8193T, 1:1000 dilution), β-actin (Cell Signaling Technology, 4970S, 1:2000 dilution), Vimentin (Abcam, Cambridge, MA, USA; ab137321, 1:1000 dilution), HA (Abcam, ab137321, 1:2000 dilution), HIS (Abcam, ab18184, 1:2000 dilution), Myc (Abcam, ab9106, 1:2000 dilution), Flag (Sino Biological, Beijing, China; 109143-MM13, 1:2000 dilution), Histone H3 (Cell Signaling Technology, 14269S, 1:1000 dilution), JNK2 (Abcam, ab236111, 1:1000 dilution), PAK1 (Cell Signaling Technology, 2602S, 1:1000 dilution), and HRP-linked Antibody (Cell Signaling Technology, 7076S or 7074S, 1:5000 dilution). After incubation, the membranes were probed with secondary antibodies, including NDR1/2 (Phospho-Thr444/442) (Affinity, Cincinnati, USA; AF8174, 1:1000 dilution), Phospho-β-catenin (Ser33/37/Thr41) (Cell Signaling Technology, 2009, 1:1000 dilution), Phospho-threonine (Cell Signaling Technology, 1:1000 dilution), and Phospho-serine (Signal way, Baltimore, USA; 1:1000 dilution). The chemiluminescent signal was detected using the Super ECL Western Blotting Detection Kit (Merck, Kenilworth, New Jersey).

### 2.17 Dosage of the NDR1 activator

Both *in vitro* cell experiments and *in vivo* animal experiments used a concentration of 5 μM for the NDR1 activator, with an administered dose of 0.1 ml per mouse in the animal experiments.

### 2.18 Statistical analysis

Statistical analyses were conducted utilizing GraphPad Prism 8 software (GraphPad, San Diego, CA, USA). All experiments were performed at least in triplicates, and quantitative data are expressed as mean ± standard deviation. Group comparisons were assessed through either one-way analysis of variance (ANOVA) or independent samples t-test, as appropriate. Statistical significance was set at P < 0.05.

## 3. Results

### 3.1 NDR1 overexpression inhibits EMT in prostate cancer

**Figure 1:**
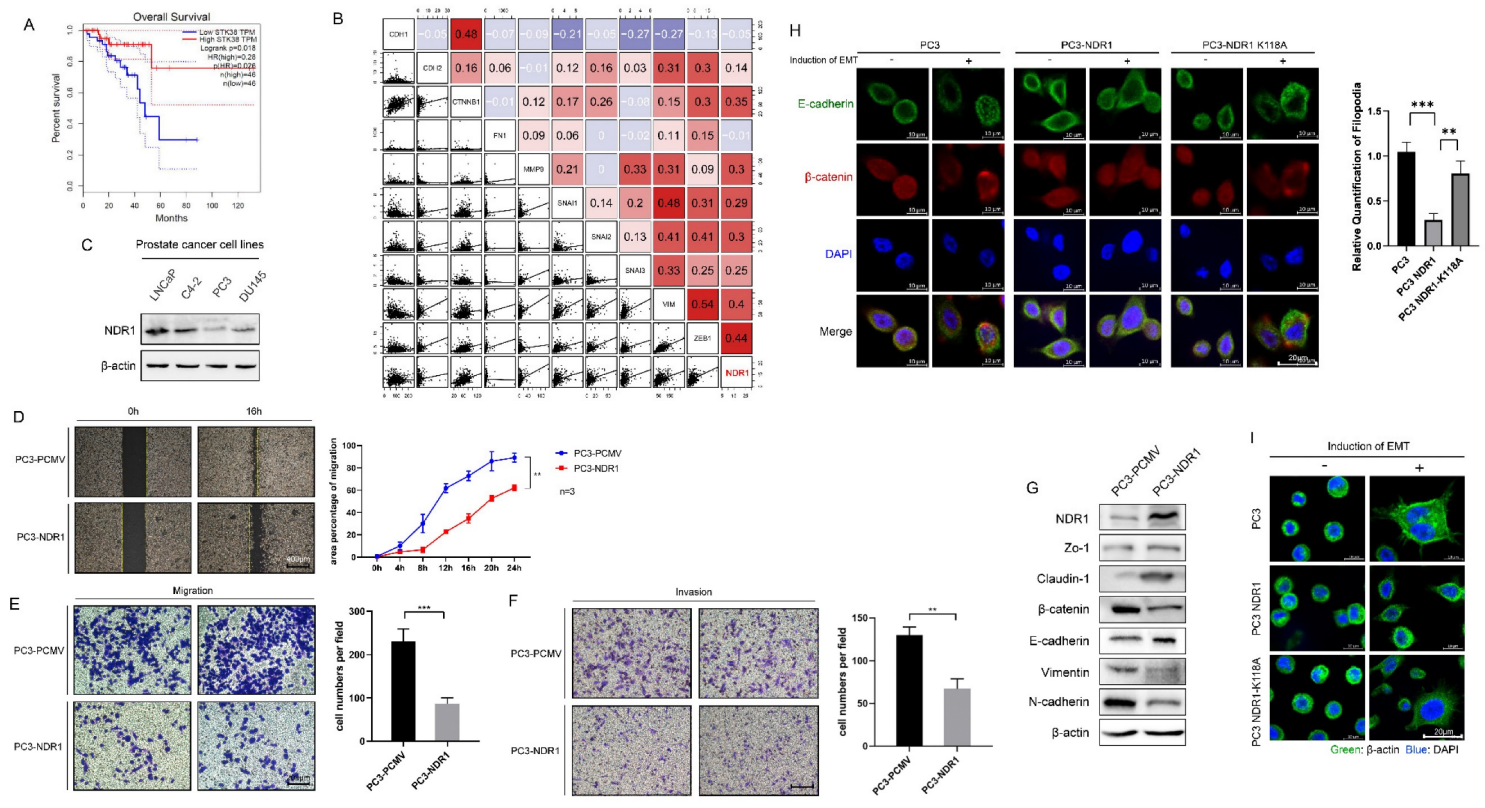
** NDR1 inhibits the EMT in the prostate cancer context. A:** The expression level of NDR1 influences the prognosis of prostate cancer patients, with poor prognosis observed in patients with low NDR1 expression. **B:** Correlation analysis revealing a significant association between the expression levels of NDR1 and EMT markers, indicating a significant correlation between NDR1 expression and the EMT markers. **C:** NDR1 expression in various prostate cancer cell lines. **D:** Overexpression of NDR1 inhibits prostate cancer cell (PC3) scratch wound healing. Scar bar = 400 μm. **E:** NDR1 inhibition suppresses PC3 cell migration. Scar bar = 200 μm. **F:** NDR1 inhibition reduces PC3 cell invasion. Scar bar = 200 μm. **G:** NDR1 reduces the expression of EMT markers (ZO-1, Claudin-1, β-catenin, E-cadherin, Vimentin, and N-cadherin) in PC3 cells. **H:** The impact of NDR1 and NDR1 (K118A) on the EMT in PC3 is characterized by E-cadherin membrane disruption and internalization. NDR1 inhibits the EMT, while NDR1 (K118A) exerts no significant effect. Scar bar = 20 μm. **I:** β-actin represents PC3 cell morphological changes and pseudopodia formation, indicating the EMT status. NDR1 inhibits the EMT, while NDR1 (K118A) exerts no significant effect. Scar bar = 200 μm. The blots and gels were cropped. The mean ± SEM values are reported (n = 3). Statistical significance was determined using a two-tailed Student's t-test, with **P < 0.01 and ***P < 0.001.

The results reveal a significant association between NDR1 expression levels and prostate cancer patient prognosis, indicating that lower NDR1 expression correlates with notably shorter survival times (**Fig. 1A**). Additionally, a significant correlation between NDR1 expression levels and certain clinical parameters of prostate cancer. Specifically, patients with low NDR1 expression tend to have a higher T stage (T3), higher N stage (N1), and higher Gleason score (8 or 9), suggesting a more advanced disease stage and poorer prognosis compared to patients with high NDR1 expression. Conversely, there were no significant differences in M stage (metastasis) or PSA level between the two groups (**Supplementary Table S2**). Further analysis revealed a significant correlation between the level of NDR1 and the levels of various proteins related to the EMT (**Fig. 1B**). Investigation of NDR1 expression in various prostate cancer cell lines revealed that NDR1 is downregulated in PC3 and DU145 cell lines, which exhibit a higher degree of malignancy (**Fig. 1C**). Further experiments demonstrated that NDR1 overexpression significantly reduced the migratory and invasive capacities of prostate cancer cells in scratch wound assays (**Fig. 1D, Fig. S1A**), migration assays (**Fig. 1E, Fig. S1B**), and invasion assays (**Fig. 1F**). Western blot analysis showed that NDR1 overexpression increased the expression level of ZO-1, Claudin-1 and E-cadherin and reduced the expression levels of β-catenin, Vimentin, and N-cadherin (**Fig. 1G**). Overall, these changes in protein expression patterns indicate a potential reversal or inhibition of the EMT process, which is often associated with increased cell motility, invasiveness, and metastatic potential in cancer cells.

To further investigate the relationship between NDR1 kinase activity and its inhibitory effect on the EMT, we generated a plasmid carrying an NDR1 variant with an ATP-binding site mutation (NDR1 K118A). A significant reduction in the phosphorylation level of NDR1 K118A (**Fig S1C**) was found, indicating that the mutation at position 118A may have disrupted the normal phosphorylation pattern of NDR1, altering its ability to interact with other cellular components involved in signaling pathways or cellular processes. Specifically, NDR1 overexpression markedly inhibited the E-cadherin membrane disruption and endocytosis induced by the EMT inducer (Transforming growth factor-beta, [TGF-β]), and this effect was significantly reduced in the presence of kinase-deficient NDR1 (**Fig. 1H**). Similarly, NDR1 and NDR1 K118A showed significant differences in their inhibitory effects on pseudopodia formation in PC3 cells after EMT induction (**Fig. 1I**). These findings confirm that NDR1 kinase activity is crucial for inhibiting EMT, as evidenced by the significant reduction in the inhibitory effect on EMT-related processes observed with the kinase-deficient NDR1 variant (NDR1 K118A).

### 3.2 β-catenin is ubiquitinated by NDR1 through the action of FBXO11

**Figure 2:**
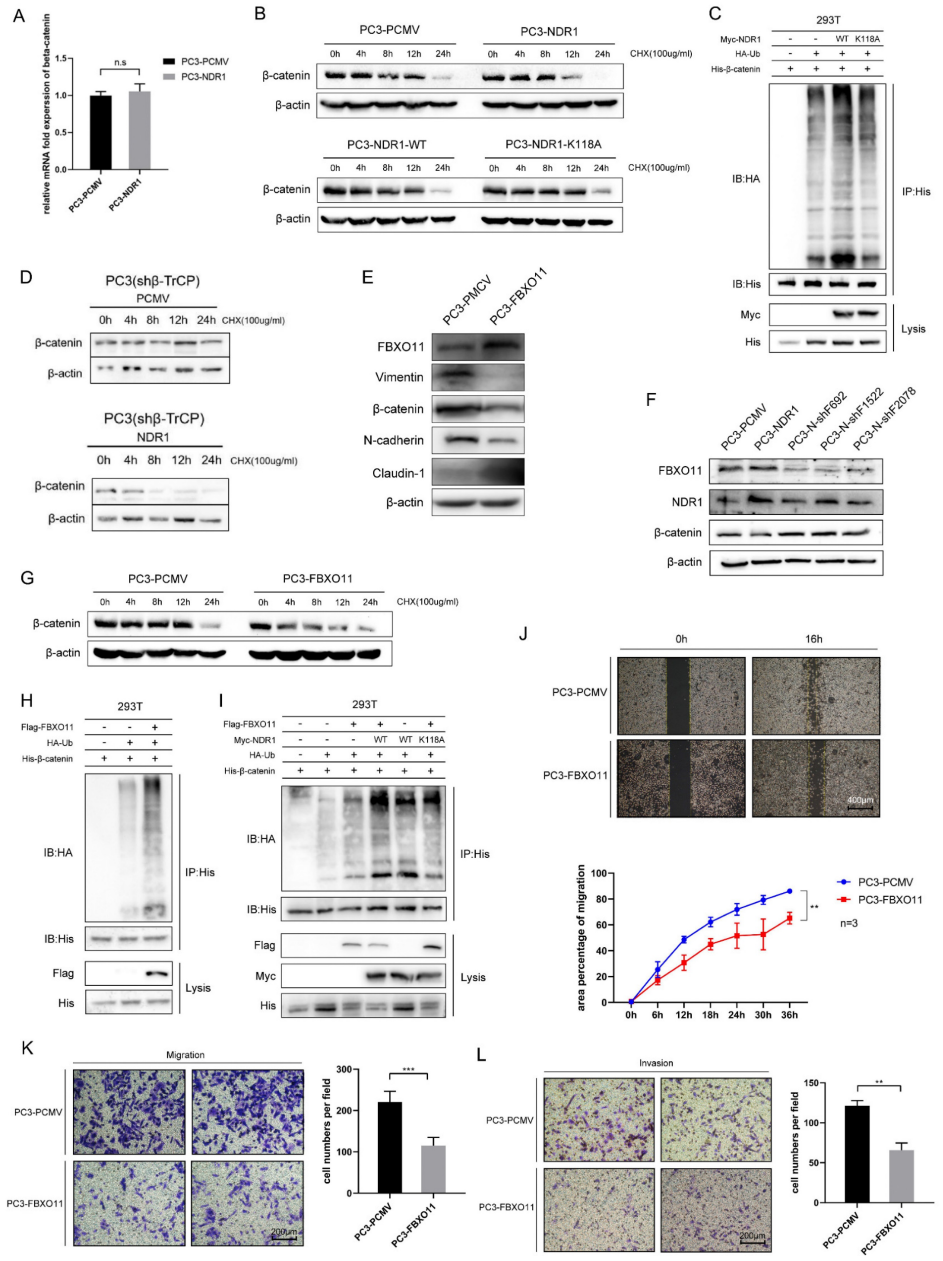
** β-catenin is ubiquitinated by NDR1 through the action of FBXO11. A:** RT-PCR for assessing the regulatory effect of NDR1 on β-catenin mRNA. **B:** Protein stability experiments using CHX to investigate the impact of wild-type NDR1 and NDR1 K118A on β-catenin protein stability. **C:** Effects of NDR1 and NDR1 K118A on β-catenin ubiquitination.**D:** Assessment of the impact of NDR1 on β-catenin protein stability upon β-TrCP knockout. **E:** Western blot analysis of the effects of FBXO11 overexpression on EMT markers in PC3. **F:** Western blot analysis of the effects of FBXO11 knockdown on NDR1 and β-catenin expression. **G:** The impact of FBXO11 on β-catenin protein stability following treatment with CHX. **H:** Effect of FBXO11 on β-catenin ubiquitination levels. **I:** Effect of FBXO11, wild-type NDR1, and NDR1 K118A on β-catenin ubiquitination levels. **J:** Cell scratch assay to investigate the impact of FBXO11 on PC3 cell wound healing ability. Scar bar = 400 μm. **K:** Cell migration assay to assess the effect of FBXO11 on PC3 cell migration ability. Scar bar = 200 μm. **L:** Cell invasion assay to examine the influence of FBXO11 on PC3 cell invasion capability. Scar bar = 200 μm. The blots and gels were cropped. The mean ± SEM values are reported (n = 3). Statistical significance was determined using a two-tailed Student's t-test, with ns P > 0.05, **P < 0.01, and ***P < 0.001.

Previously, we found that NDR1 significantly inhibits the expression of β-catenin. Building upon this, we observed that NDR1 overexpression does not affect the mRNA levels of β-catenin (**Fig. 2A**); thus, the impact of NDR1 on β-catenin mRNA levels could be deduced to be minimal. Protein stability experiments revealed that NDR1 overexpression significantly reduced the stability of β-catenin after cycloheximide (CHX) treatment, while NDR1 K118A exerted no such effect (**Fig. 2B, Fig. S1E**). Specifically, Comparing PC3-PCMV and PC3-NDR1, in lanes treated with cycloheximide (CHX) for 12 hours, the expression of β-catenin was found to be significantly lower in the NDR1 overexpression group, while in lanes treated with CHX for 24 hours, the NDR1 overexpression group shows no expression of β-catenin, while the control group PCMV still expresses β-catenin. When NDR1 is mutated to the kinase-inactive mutant NDR1 K118A, there was no significant difference in β-catenin expression, demonstrating that NDR1's effect on β-catenin protein stability could indeed be depending on NDR1's kinase activity.

Protein degradation in cells is mainly mediated through the ubiquitin-proteasome pathway and autophagy. We measured the ubiquitination rate of β-catenin and found that wild-type NDR1 significantly promoted β-catenin ubiquitination, while NDR1 K118A exerted no such effect (**Fig. 2C**). These results suggest that the effect of NDR1 on β-catenin levels is not mediated through changes in β-catenin mRNA expression. Instead, NDR1 overexpression decreases β-catenin stability by promoting its ubiquitination and subsequent degradation via the ubiquitin-proteasome pathway, while the K118A mutation in NDR1 abolishes this effect, indicating the importance of NDR1 kinase activity in regulating β-catenin stability.

Previous studies have shown that the ubiquitination and degradation of β-catenin depend on the E3 ubiquitin ligase β-TrCP 37. However, we found that the effect of NDR1 in reducing β-catenin protein stability remained intact after β-TrCP was knocked down (**Fig. 2D, Fig. S1F**), and a mass spectrometry (MS) analysis of the molecules bound to NDR1 and β-catenin revealed that these proteins interacted with the E3 ubiquitin ligase FBXO11. Furthermore, IP assays were performed to confirm the interaction between FBXO11, NDR1, and β-catenin (**Fig. S1H-J**). Therefore, we speculated that NDR1 regulates β-catenin ubiquitination through a non-canonical pathway, possibly involving the E3 ubiquitin ligase FBXO11, rather than the canonical pathway involving β-TrCP.

To demonstrate the role of FBXO11 in regulating β-catenin degradation, a series of experiments were conducted. The results revealed that overexpression of FBXO11 significantly suppressed β-catenin protein expression (**Fig. 2E**), whereas knockdown of FBXO11 (**Fig. S1N**) led to a significant increase in β-catenin levels (**Fig. 2F**). FBXO11 overexpression notably reduced β-catenin protein stability after treatment using CHX (**Fig. 2G, Fig. S1G**) and increased its ubiquitination levels (**Fig. 2H**). Co-overexpression of wild-type NDR1 and FBXO11 exhibited a significantly greater positive effect on β-catenin ubiquitination compared to that induced the overexpression of either protein or the overexpression of both NDR1 K118A and FBXO11 (**Fig. 2I**). Consistent with the EMT-promoting effect of NDR1, FBXO11 overexpression significantly suppressed the healing, migration and invasion of PC3 cells (**Fig. 2J-L**). Taken together, these findings indicate that NDR1 ubiquitinates β-catenin through FBXO11independent of the canonical pathway and thereby inhibits the EMT in the context of prostate cancer.

### 3.3 NDR1 phosphorylates FBXO11 at Ser187

**Figure 3:**
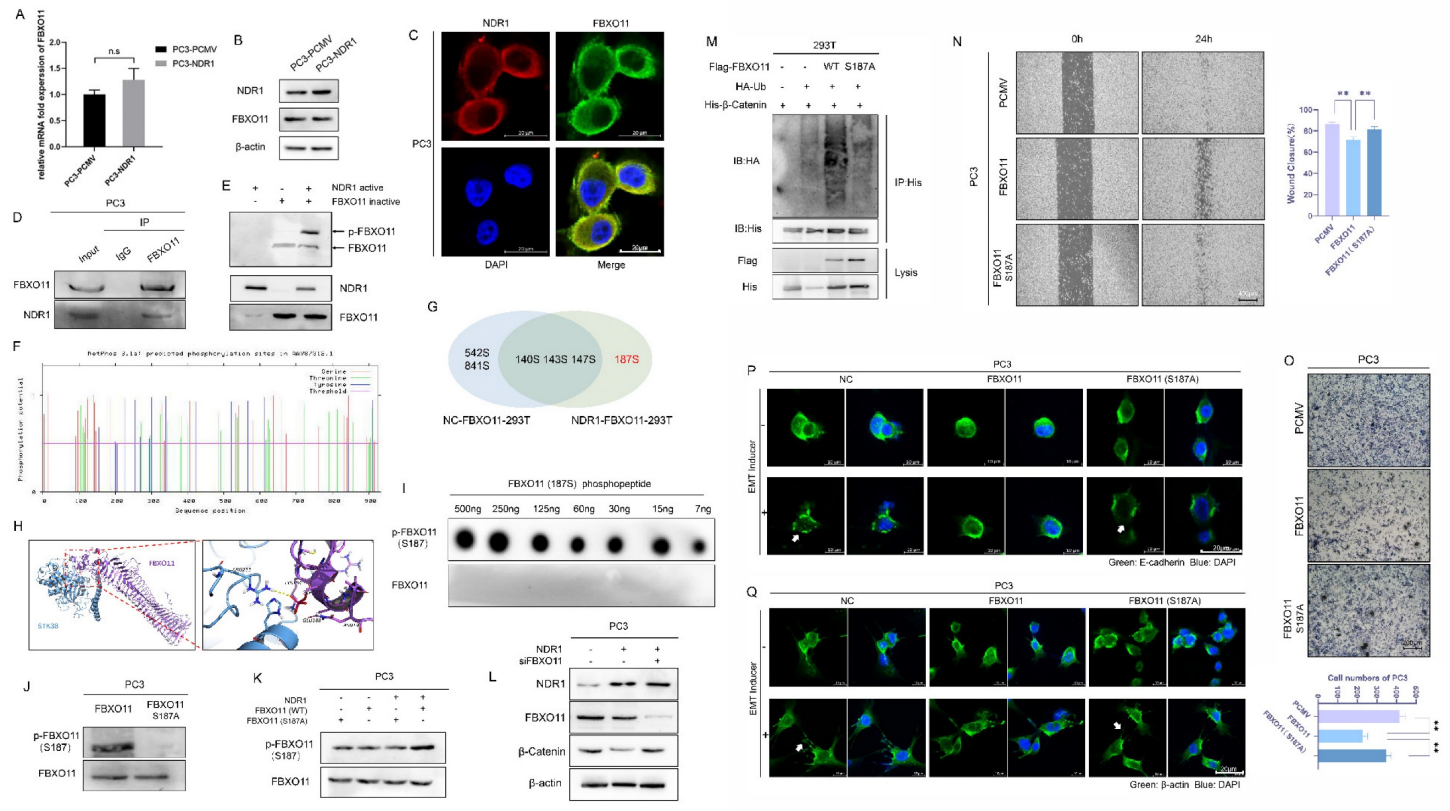
** NDR1 phosphorylates FBXO11 at Ser187. A:** RT-PCR to investigate the regulatory effect of NDR1 on FBXO11 mRNA. **B:** Western blot experiment rules out the regulatory effect of NDR1 on FBXO11 mRNA. **C:** Immunofluorescence (IF) assay confirms the colocalization of NDR1 and FBXO11 in subcellular compartments, providing spatial evidence of their mutual interaction. Scar bar = 20 μm. **D:** IP with endogenous proteins confirms the mutual interaction between NDR1 and FBXO11. **E:** Phos-tag assay reveals *in vitro* NDR1-mediated phosphorylation of FBXO11. **F:** Net-Phos3.1 was used to predict the FBXO11 sites phosphorylated by NDR1. **G:** Tandem phosphorylation mass spectrometry results. **H:** Docking analysis predicts the binding interaction between NDR1 and FBXO11 and corresponding binding sites. **I:** The designed FBXO11 S187 phosphorylated peptide and a synthesized polyclonal antibody (rabbit) were used for the dot-blot analysis performed to establish the specificity of the antibody to p-FBXO11 S187. **J:** An inactive FBXO11 (S187A) mutant was constructed; the phosphorylation antibody did not recognize the inactive mutation site in PC3. **K:** Verification of the NDR1 phosphorylation of FBXO11 (WT) and FBXO11 (S187A); NDR1 phosphorylation of the inactivated mutant was significantly reduced. **L:** After FBXO11 knockout, NDR1 loses its inhibitory effect on β-catenin protein expression. **M:** After the inactivation mutation was introduced, FBXO11 ubiquitination of β-catenin was significantly inhibited. **N, O:** After the inactivation mutation was introduced, the FBXO11 inhibitory effect on PC3 scratch wound healing and migration was significantly reduced. N Scar bar = 400 μm; O Scar bar = 200 μm. **P, Q:** After the inactivation mutation was introduced, FBXO11 inhibitory effects on PC3 E-cadherin membrane localization disruption, endocytosis, and pseudopodia formation were significantly reduced. Scar bar = 20 μm. The blots and gels were cropped. The mean ± SEM values are reported (n = 3). No significant difference was observed (ns P > 0.05) according to a two-tailed Student's t-test.

NDR1 is a serine/threonine kinase 38 that exerts regulatory effects on the EMT in prostate cancer through its kinase activity. To explore whether NDR1 functions by phosphorylating FBXO11, herein, we conducted further investigations. NDR1 overexpression did not alter the RNA or protein expression levels of FBXO11 (**Fig. 3A-B**). IF assay results indicated that NDR1 and FBXO11 colocalized in cellular subspaces, providing spatial evidence for their interaction (**Fig. 3C**). Immunoprecipitation experiments with both endogenous and exogenous proteins confirmed the binding of NDR1 and FBXO11 (**Fig. 3D, Fig. S1J**).

To investigate the phosphorylation of FBXO11 by active NDR1, we utilized the phosphorylation-specific gel dye Phos-tag, which enhances the interaction between phosphorylated proteins and the gel, leading to slower migration of phosphorylated proteins during electrophoresis. With this method, we detected significant activation of FBXO11 by active NDR1 in the presence of ATP (**Fig. 3E**). To identify the site in FBXO11 phosphorylated by NDR1, we employed NetPhos3.1 39, which enables the prediction of phosphorylation sites, and those with scores higher than 0.5 were selected (**Fig. 3F, Table S3**). Using liquid chromatography (LC)‒MS/MS combined with a phosphorylation-specific enrichment strategy, we analyzed phosphorylation events in samples of proteins overexpressing FBXO11 alone, as well as those coexpressing NDR1 and FBXO11 (**Fig. S2, Fig. S3**and **Table S4**). We found a high frequency of FBXO11 phosphorylation at Ser187 only in samples coexpressing NDR1 and FBXO11 (**Fig. 3G**). Notably, the NetPhos3.1 score for this phosphorylation site was a high score of 0.956 (**Table S3**). Docking prediction results showed that Ser187 in FBXO11 forms a hydrogen bond with Arg211 in NDR1, resulting in the binding of these proteins (**Fig. 3H**). To validate the specificity of FBXO11 phosphorylation at Ser187, we synthesized an FBXO11 S187-phosphorylated peptide to generate a specific phosphorylation antibody. Dotblot assays confirmed the specific recognition of this antibody for the phosphorylated FBXO11 peptide (**Fig. 3I**).

To validate the functional significance of FBXO11 phosphorylation at Ser187, we generated an inactivating mutation, FBXO11 S187A, by substituting the serine residue with a non-phosphorylatable alanine residue (**Fig. 3J**). Then, we used overexpressed wild-type FBXO11 (WT), a mutant FBXO11 with a phosphorylation site mutation at S187 (S187A) to prevent phosphorylation at S187, co-overexpression of NDR1 and mutant FBXO11, and co-overexpression of NDR1 and wild-type FBXO11 (**Fig. 3K**). In addition, we also used a specific phosphorylation antibody against FBXO11 S187 to detect the phosphorylation status at the FBXO11 S187 site. The Western blot analysis results showed that NDR1 overexpression with the presence of wild-type FBXO11 significantly promoted the phosphorylation of FBXO11 at S187, counteracting the effects of FBXO11 S187A with NDR1 overexpression on the phosphorylation of FBXO11 at S187 and supporting a synergy between NDR1 and FBXO11 (**Fig. 3K**). Additionally, wild-type FBXO11 markedly increased the β-catenin ubiquitination rate, an effect that was lost with FBXO11 S187A (**Fig. 3M**). The observed phenomenon of weakened binding between FBXO11 and β-catenin may lead to reduced ubiquitination of β-catenin due to the presence of the FBXO11 S187A mutation (**Fig. S1O**). In addition, the inactivating mutation FBXO11 S187A failed to promote the acquisition of various phenotypes characteristic of the EMT in prostate cancer; for example, compared to FBXO11 overexpression, it induced scratch wound closure (**Fig. 3N**), cell migration (**Fig. 3O**), E-cadherin membrane disruption and endocytosis (**Fig. 3P**), and pseudopodia formation (**Fig. 3Q**). The rescue experiments performed after FBXO11 was knocked out (**Fig. 3L**) showed that NDR1 failed to exert an inhibitory effect on β-catenin protein expression, indicating that NDR1 regulates β-catenin through the action of FBXO11. In summary, these findings confirm that NDR1 phosphorylates FBXO11 at Ser187, and this phosphorylation-mediated FBXO11 ubiquitination of β-catenin regulates the EMT and prostate cancer progression.

### 3.4 NDR1 promotes β-catenin ubiquitination by phosphorylating β-catenin at Ser33/37

**Figure 4:**
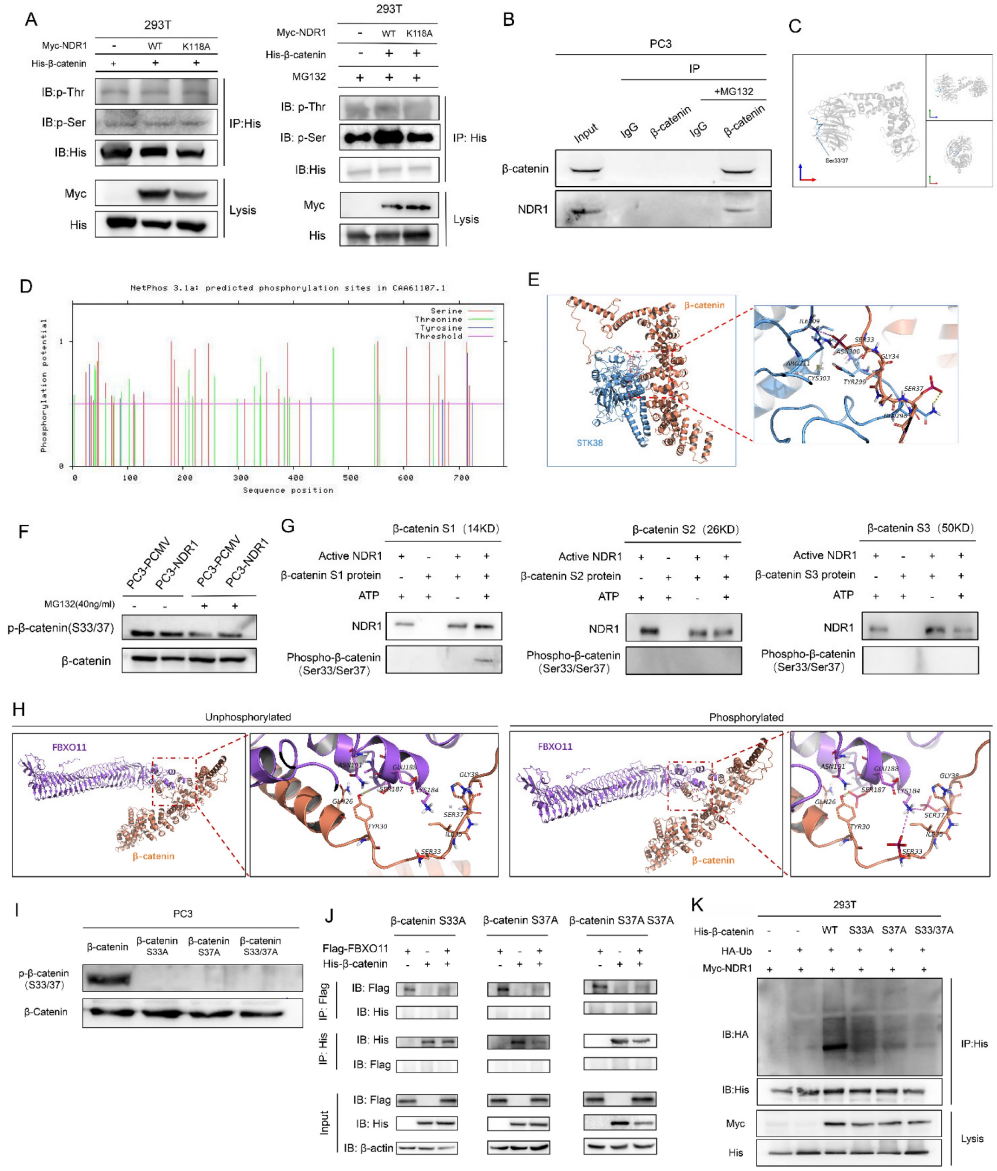
** NDR1 promotes β-catenin ubiquitination by phosphorylating β-catenin at Ser33/37. A:** The panphosphorylation levels of IP-enriched β-catenin were measured, showing that NDR1 exerted no significant effect on β-catenin phosphorylation levels. Inhibition of proteasome by MG132 followed by IPenrichment of β-catenin revealed that NDR1 significantly promoted β-catenin phosphorylation. **B:** IP with endogenous proteins confirmed the interaction between NDR1 and β-catenin. NDR1 and β-catenin bind after ubiquitination is inhibited. **C:** Schematic representation of the protein spatial structure of β-catenin. **D:** NetPhos3.1 was used to predict sites of NDR1 phosphorylation in β-catenin. **E:** Docking analysis predicts the binding interaction between NDR1 and β-catenin and corresponding binding sites. **F:** After MG132 treatment, NDR1 phosphorylation of β-catenin at S33/37 was increased compared to that of the control PC3-PCMV. **G:** The β-catenin truncation mutant; an *in vitro* kinase assay confirmed that NDR1 phosphorylated S33/37 in the S1 region without phosphorylating sites in the S2 and S3 region. **H:** Docking prediction of the binding affinity and corresponding sites of β-catenin with FBXO11 before and after β-catenin phosphorylation. **I:** Construction of β-catenin phosphorylation site mutants (S33A, S37A, and S33/37A) and validation using phosphorylation-specific antibodies. **J:** Plasmids carrying β-catenin with S33A and S37A single-point mutations or S33A-37A double-point mutations were constructed to perform IP with exogenous proteins, and the results showed that the point mutations abolished the binding of β-catenin to FBXO11. **K:** After β-catenin S33A, S37A single-point, and S33A-37A double-point mutations were introduced, the NDR1-mediated increase of β-catenin ubiquitination was significantly reduced. The blots and gels were cropped.

To investigate the effect of NDR1 phosphorylation on β-catenin, we examined the serine phosphorylation status of β-catenin in samples through IP. The results showed that in the presence of the proteasome inhibitor MG132 40, NDR1 significantly increased the phosphorylation rate of a β-catenin serine residue, while no significant difference in the phosphorylation rate of β-catenin in samples expressing the NDR1 K118A mutant and in the absence of a ubiquitination inhibitor (**Fig. 4A**). IP with exogenous proteins demonstrated the binding of NDR1 and β-catenin (**Fig. S1I**), while in an IP assay with endogenous proteins, an interaction signal was observed only after MG132 treatment (**Fig. 4B**). This finding suggests that the NDR1 and β-catenin binding and NDR1 phosphorylation of β-catenin are associated with β-catenin ubiquitination. We hypothesized that NDR1 phosphorylation of β-catenin may lead to its ubiquitination and degradation. Thus, after ubiquitination was inhibited, phosphorylation and binding were retained. Furthermore, we predicted the sites of β-catenin that are phosphorylated by NDR1 using NetPhos3.1^23^ (**Fig. 4D, Supplementary Table S5**), and a docking simulation revealed that β-catenin Ser33 forms a salt bridge with NDR1 Arg211 and that Ser37 forms a hydrogen bond with β-catenin Gln296 (**Fig. 4E**). Using various phosphorylation-specific antibodies, we detected the phosphorylation of Ser33/37 by NDR1 after MG132 treatment (**Fig. S1L**). Ser33/37 is located in the peripheral region of the β-catenin protein structure, where it can easily interact with other proteins (**Fig. 4C**). The inhibition of ubiquitination significantly increased NDR1-mediated phosphorylation of β-catenin Ser33/37 compared to the control PC3-PCMV (**Fig. 4F**). These serine residues are located in the N-terminal domain of β-catenin 41. Considering the location of these sites, we generated truncated forms of β-catenin, namely, S1-14KD (K19-T150), S2-26KD (R151-S318), and S3-50KD (G319-L782). An *in vitro* kinase assay confirmed the effect of active NDR1 phosphorylation on the S1-truncated form of β-catenin in the presence of ATP, while no phosphorylation of the other truncated forms was detected (**Fig. 4G**).

The protein-binding interaction between FBXO11 and β-catenin was predicted using a docking analysis. It was found that FBXO11 and β-catenin did not bind prior to β-catenin phosphorylation. However, after β-catenin phosphorylation, hydrogen bonds were formed between β-catenin Ser33, Ser37, and FBXO11 Lys184 and between β-catenin Tyr30 and FBXO11 Ser187 (**Fig. 4H**). These findings suggest that β-catenin phosphorylation promotes its binding to FBXO11. To further confirm the functionality of the phosphorylation sites in β-catenin, Ser33 and Ser37 were subjected to site-directed mutagenesis by constructing β-catenin phosphorylation site mutants (S33A, S37A and S33/37A), which resulted in the loss of phosphorylation signals at these sites (**Fig. 4I**). The binding of β-catenin and FBXO11 was subsequently abolished by these mutations (**Fig. 4J**), and the increased ubiquitination of β-catenin mediated by NDR1 was attenuated (**Fig. 4K**). In conclusion, these results demonstrate that β-catenin is phosphorylated at S33/37 by NDR1, which drives the binding of β-catenin to FBXO11, causing β-catenin ubiquitination and subsequent degradation.

### 3.5 NDR1-FBXO11 inhibits β-catenin nuclear translocation through JNK2 ubiquitination

**Figure 5:**
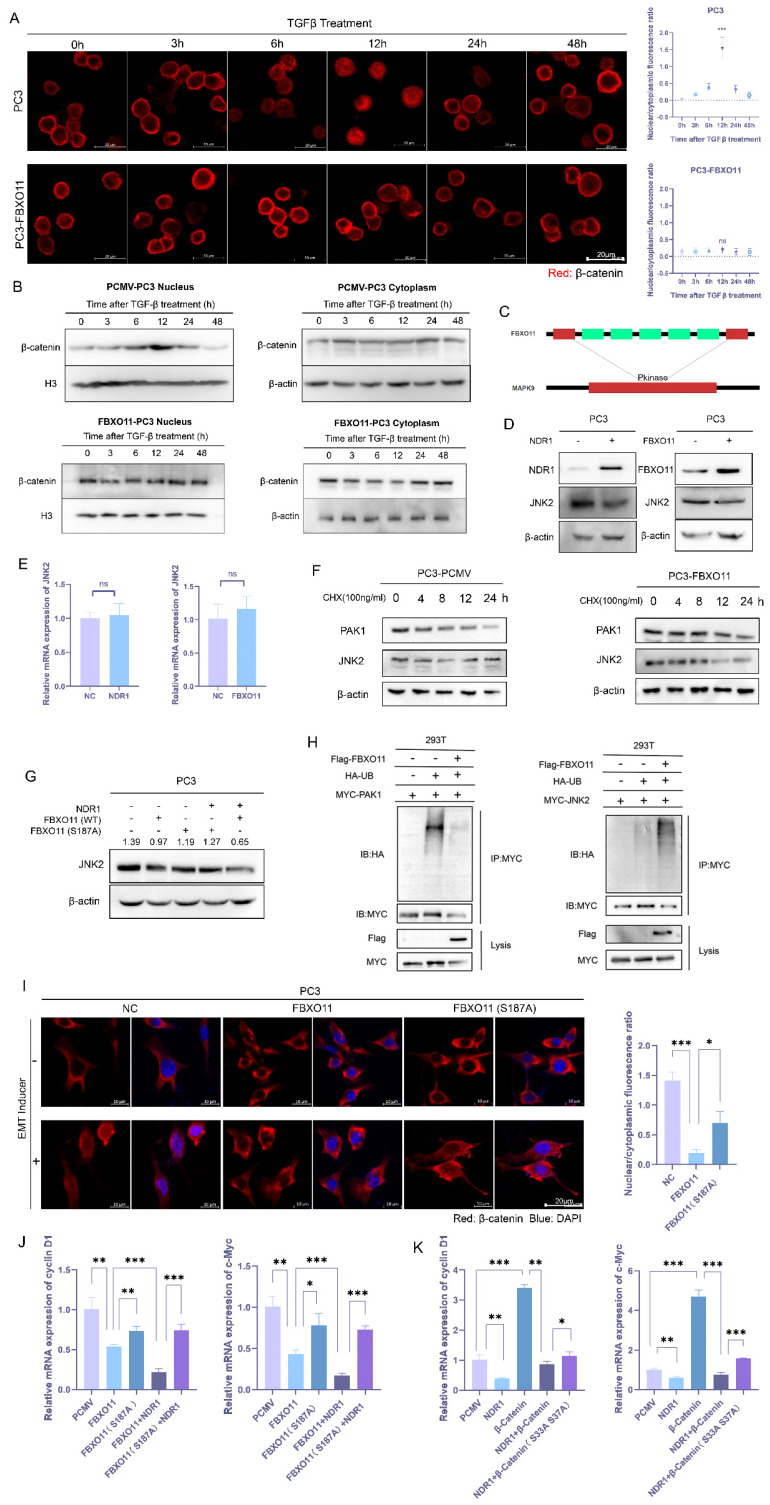
** NDR1-FBXO11 inhibits β-catenin nuclear translocation through JNK2 ubiquitination. A:** Following the TGF-β-induced EMT, a time-dependent immunofluorescence (IF) analysis revealed β-catenin nuclear translocation. Scar bar = 20 μm. **B:** The protein levels in different cell fractions (proteins in the cell membrane, nucleus, and cytoplasm) were measured to validate β-catenin nuclear translocation and the effect of FBXO11 on this process. **C:** FBXO11 was predicted to ubiquitinate proteins related to β-catenin nuclear translocation, such as JNK2. **D, E:** NDR1 and FBXO11 do not affect JNK2 mRNA levels but significantly reduce JNK2 protein expression. **F:** FBXO11 reduces the protein stability of PAK1 and JNK2, with JNK2 more significantly affected. **G:** NDR1 and FBXO11 reduced JNK2 protein levels, while FBXO11 S187A exerts no significant effect on JNK2 protein levels. **H:** FBXO11 ubiquitinates JNK2, while PAK1 is not ubiquitinated. **I:** FBXO11 inhibits β-catenin nuclear translocation, while FBXO11 (S187A) could reverse this effect. Scar bar = 20 μm. **J, K:** RT-PCR analysis of cyclin D1 and c-Myc mRNA levels under the influence of NDR1, FBXO11, β-catenin, and their respective mutations. The blots and gels were cropped. The mean ± SEM values are reported (n = 3). Statistical significance is represented as ns P>0.05, *P<0.05, **P < 0.01, ***P < 0.001 according to a two-tailed Student's t-test.

The transcriptional activity of β-catenin depends on its nuclear translocation. However, after the EMT was induced, the nuclear localization of β-catenin was inhibited (**Fig. 1H**). Previous studies have shown that β-catenin, after exerting a transcriptional effect, is regulated via a complex transcriptional termination mechanism. Thus, β-catenin is either degraded within the nucleus or exported through nuclear pore complexes (NPCs) 42-45. To investigate the dynamics of β-catenin nuclear translocation, we conducted time-course IF assays. The results revealed that TGF-β stimulation led to time-dependent fluctuations in the nuclear signal intensity of β-catenin, with an initial signal increase followed by a decrease (**Fig. 5A**, upper panel). However, FBXO11 overexpression significantly inhibited the nuclear translocation of β-catenin (**Fig. 5A**, lower panel). We also performed Western blot experiments with proteins in different cell fractions to validate these findings. The data demonstrated consistent fluctuations in nuclear β-catenin expression after TGF-β stimulation, while cytoplasmic β-catenin expression was initially slightly decreased and then increased (**Fig. 5B**, upper panel). These findings suggest that the increase in nuclear β-catenin may be due to its nuclear translocation from the cytoplasm. The considerable disparity in cytoplasmic and nuclear protein abundance may contribute to the inconsistent changes observed. Notably, the expression of membrane-bound β-catenin followed no particular pattern (**Fig. S1M**), ruling out the possibility that changes in β-catenin expression are caused by the dissociation of membrane-bound proteins. Consistently, overexpression of FBXO11 abrogated the aforementioned outcomes (**Fig. 5B**, lower panel).

To explore the mechanism by which FBXO11 regulates β-catenin nuclear translocation, ubiquitination substrates of FBXO11 were predicted using UbiBrowser 46, and the potential substrates included c-Jun N-terminal kinase 2 (JNK2) and p21 activated kinase 1 (PAK1) (**Fig. 5C, Fig. S4D**). Previous studies have reported that JNK2 is activated by Rac1 and phosphorylates β-catenin at S191 and S605, promoting its nuclear translocation 47, while PAK1 interacts with β-catenin to regulate its nuclear translocation 48. Subsequent experiments revealed that FBXO11 significantly reduced JNK2 protein stability and promoted its ubiquitination, while its impact on PAK1 protein stability or ubiquitination was not as significant (**Fig. 5F, H**). The expression of NDR1 and FBXO11 significantly reduced JNK2 protein expression (**Fig. 5D**) without affecting its mRNA levels (**Fig. 5E**), suggesting that NDR1 and FBXO11 may regulate JNK2 protein stability or degradation post-transcriptionally. The overexpression of NDR1 with FBXO11 wild type synergistically reduced the protein level of JNK2, and the effect of FBXO11 S187A on JNK2 protein level was not significant (**Fig. 5G**). Similarly, compared to wild-type FBXO11, FBXO11 S187A could significantly counteract the inhibitory effect on β-catenin nuclear translocation (**Fig. 5I**).

After entering the nucleus, β-catenin interacts with other transcription factors, thereby activating the transcription of various downstream target genes, including cyclin-D1 and c-Myc 49. Overexpression of FBXO11 led to a significant reduction in the mRNA levels of cyclin-D1 and c-Myc, and the inactivation mutation (FBXO11 S187A) weakened this effect (**Fig. 5J**). In addition, co-overexpression of FBXO11 and NDR1 enhanced the significant reduction in the mRNA levels of cyclin-D1 and c-Myc, while the inactivation mutation of FBXO11 reversed this effect (**Fig. 5J**). Similarly, NDR1 effectively reduced the excessive transcription caused by β-catenin overexpression, and the inactivation mutation of the β-catenin phosphorylation site weakened this effect to some extent (**Fig. 5K**), demonstrating that NDR1 regulation of β-catenin depends on the S33/37 phosphorylation sites. In conclusion, these results demonstrate that FBXO11 inhibits β-catenin nuclear translocation through JNK2 ubiquitination, and this process also depends on NDR1-mediated phosphorylation of FBXO11 at S187.

### 3.6 The NDR1-FBXO11-β-catenin signaling axis regulates prostate cancer lung metastasis

**Figure 6:**
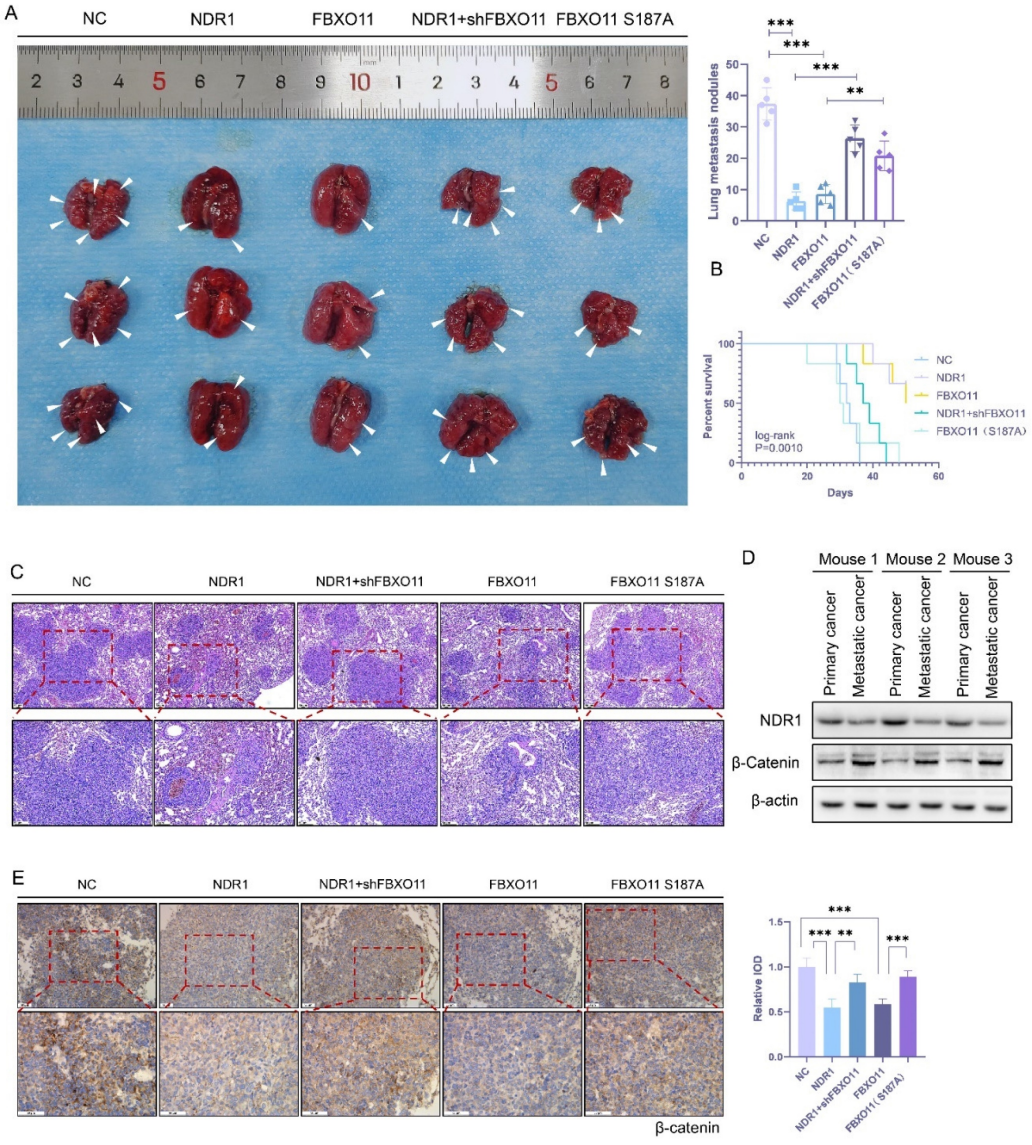
** The NDR1-FBXO11-β-catenin signaling axis regulates prostate cancer lung metastasis. A:** Representative images of lung metastasis in nude mice and the number of metastases are shown. **B:** Survival curve of tumor-bearing nude mice is presented. **C:** HE staining of lung metastatic lesions in prostate cancer-bearing nude mice is displayed. **D:** Western blot results of tumor tissues from orthotopic prostate cancer and prostate cancer lung metastatic lesions in nude mice are shown. **E:** Immunohistochemical (IHC) staining of β-catenin in lung metastatic lesions of prostate cancer-bearing nude mice, along with statistical analysis of the integrated optical density (IOD) analysis of stained tissues. The blots and gels were cropped. The mean ± SEM values are reported (n = 3). Statistical significance is indicated as **P < 0.01, ***P < 0.001 according to a two-tailed Student's t-test.

To validate the impact of the NDR1-FBXO11-β-catenin signaling axis on prostate cancer metastasis at the organism level, we constructed PC3 cell lines with NDR1 overexpressed, FBXO11 overexpressed, NDR1 overexpressed with FBXO11 knocked out, and FBXO11 phosphorylation site inactivation caused by an introduced mutation. These PC3 cell lines were intravenously injected into nude mice to establish lung metastasis models. The results showed that compared to that in the control group, overexpression of either NDR1 or FBXO11 significantly inhibited the formation of lung metastasis (**Fig. 6A**). However, when NDR1 was overexpressed and FBXO11 was knocked out, the number of metastatic foci increased significantly compared to that in the NDR1 overexpression group (**Fig. 6A**). The impact of FBXO11 S187A on metastatic foci number was also significantly weaker than that of FBXO11 overexpression (**Fig. 6A**). Furthermore, NDR1 and FBXO11 overexpression significantly prolonged the survival period of tumor-bearing nude mice, but the effect on the survival period was significantly weakened when NDR1 was overexpressed and FBXO11 was knocked out or when the FBXO11 S187A mutation was introduced (**Fig. 6B**). Histological examination of the lung tissues excised from the nude mice and stained with haemoxylin-eosin (HE) staining revealed that the sizes of lung metastatic foci were significantly smaller in the NDR1 and FBXO11 overexpression groups than in the control group (**Fig. 6C**). However, when FBXO11 was knocked due to NDR1 overexpression or when FBXO11 was mutated at the phosphorylation site, the sizes of the metastatic foci were larger than those in the NDR1-only overexpression and FBXO11 overexpression groups (**Fig. 6C**).

Next, we collected protein samples from both orthotopic prostate cancer and metastatic lesions in nude mice for Western blot analyses. The results indicated that the expression of NDR1 in the metastatic lesions was significantly lower than that in the orthotopic tumors, while the expression of β-catenin showed the opposite trend (**Fig. 6D**). Immunohistochemistry of prostate cancer lung metastatic lesions from the nude mice confirmed that NDR1 and FBXO11 overexpression suppressed the expression of β-catenin. However, the effects could be reversed when FBXO11 was knocked out or when the FBXO11 phosphorylation site was inactivated due to NDR1 overexpression (**Fig. 6E**). Collectively, these findings demonstrate the regulatory role played by the NDR1-FBXO11 signaling axis during prostate cancer metastasis. NDR1, through its interaction with FBXO11, inhibits the expression of β-catenin, and this process is dependent on NDR1 phosphorylation at S187 of FBXO11. These results elucidated the mechanism through which the NDR1-FBXO11-β-catenin axis influences prostate cancer metastasis and suggest potential therapeutic targets for managing this disease.

### 3.7 NDR1 activator inhibits prostate cancer metastasis

**Figure 7:**
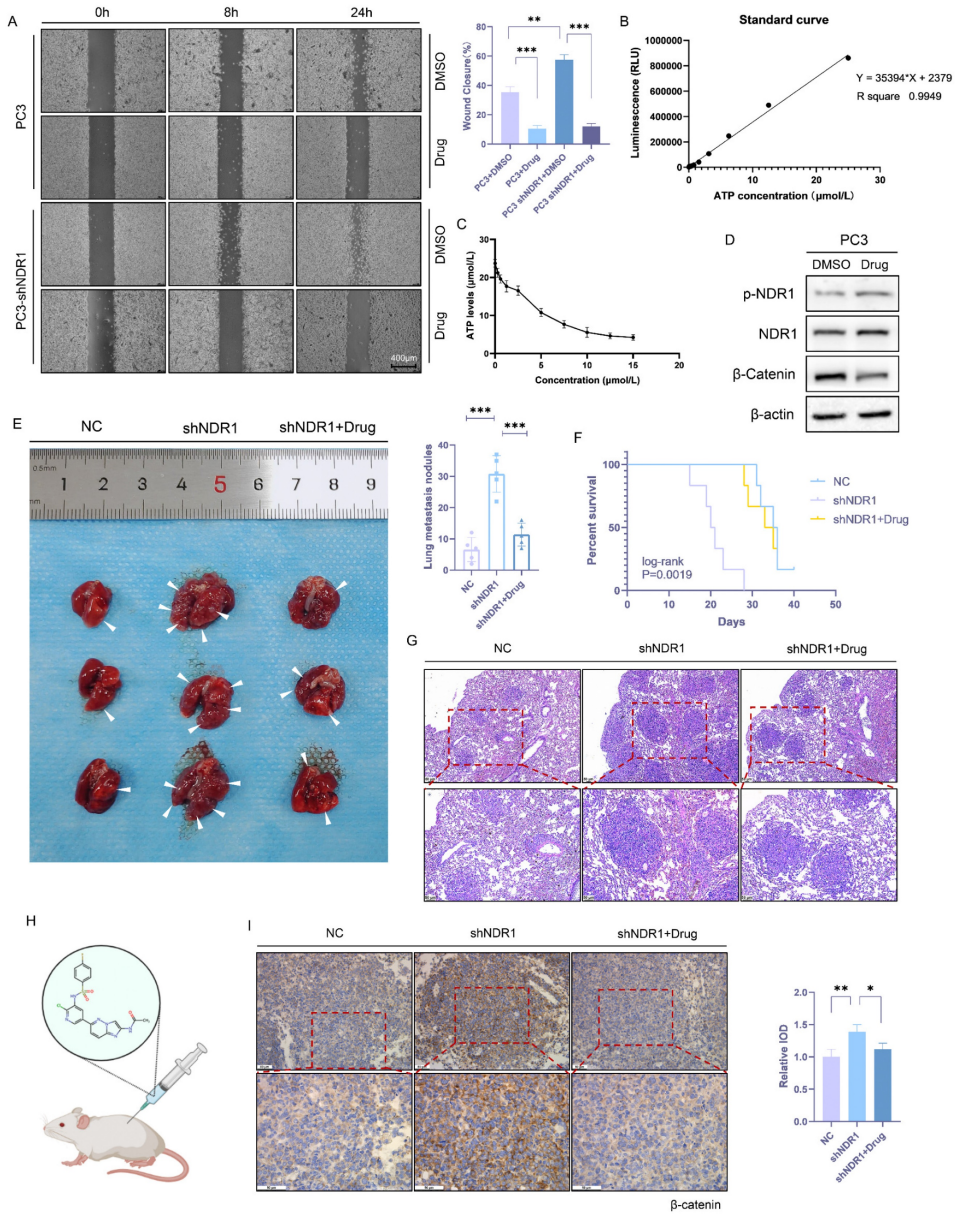
** NDR1 activator inhibits prostate cancer metastasis. A:** NDR1 knockout promotes the mobility of PC3 cells in scratch wound healing experiments, and the use of an NDR1 activator (aNDR1) inhibits these effects. **B:** Standard curve of ATP concentration and fluorescence intensity *in vitro*. **C:** ATP consumption assay demonstrates the *in vitro* activation effect of aNDR1 on NDR1 protein expressed in prokaryotes. **D:** Western blot confirming the impact of aNDR1 on the expression levels of p-NDR1, NDR1, and β-catenin. **E:** Representative images of lung metastases in nude mice and the number of metastases. **F:** Survival curve of tumor-bearing nude mice. **G:** HE staining of lung metastatic lesions in prostate cancer-bearing nude mice. **H:** Structure of aNDR1 and the administration of treatment to model mice. **I:** IHC staining of β-catenin in lung metastatic lesions of prostate cancer-bearing nude mice along with statistical analysis of the integrated optical density (IOD) of the stained tissues. The blots and gels were cropped. The mean ± SEM values are reported (n = 3). Statistical significance is indicated as *P < 0.05, **P < 0.01, ***P < 0.001 according to a two-tailed Student's t-test.

In this study, small-molecule compounds targeting NDR1 were screened from the ChEMBL database 50, and thus, an NDR1-specific activator named "aNDR1" was identified (**Fig. S4C**). *In vitro* experiments were conducted in which the inactive NDR1 protein was expressed in the presence of aNDR1 and ATP, and then, the NDR1 activation was assessed based on the amount of ATP consumed. The results demonstrated that aNDR1 exhibited concentration-dependent activation of the inactive NDR1 protein (**Fig. 7B-C**). Moreover, treatment with aNDR1 led to a significant increase in NDR1 phosphorylation and a decrease in β-catenin expression levels (**Fig. 7D**). Furthermore, *in vitro* assays revealed that aNDR1 significantly inhibited the wound healing ability of PC3 prostate cancer cells and showed an antagonistic effect against the wound healing ability that had been increased after NDR1 knockdown (**Fig. 7A**).

A lung metastasis model of prostate cancer was established with nude mice, and aNDR1 was administered subcutaneously to these mice every other day (**Fig. 7H**). The results demonstrated that aNDR1 effectively reversed the increase in the number of lung metastatic lesions caused by NDR1 knockdown (**Fig. 7E**) and significantly extended the tumor-bearing survival period of the nude mice (**Fig. 7F**). Histological examination of lung tissue sections stained with HE showed that aNDR1 significantly attenuated the increase in the size of metastatic lesions that had been induced by NDR1 knockdown (**Fig. 7G**). The IHC staining results revealed that NDR1 knockdown led to a significant upregulation of β-catenin expression, whereas aNDR1 expression markedly reduced β-catenin expression levels (**Fig. 7I**). These findings indicate that aNDR1 is a promising candidate for a small-molecule lead compound with potential clinical value. Its activation of NDR1 led to therapeutic effects on aggressive prostate cancer with low NDR1 expression.

**Figure 8:**
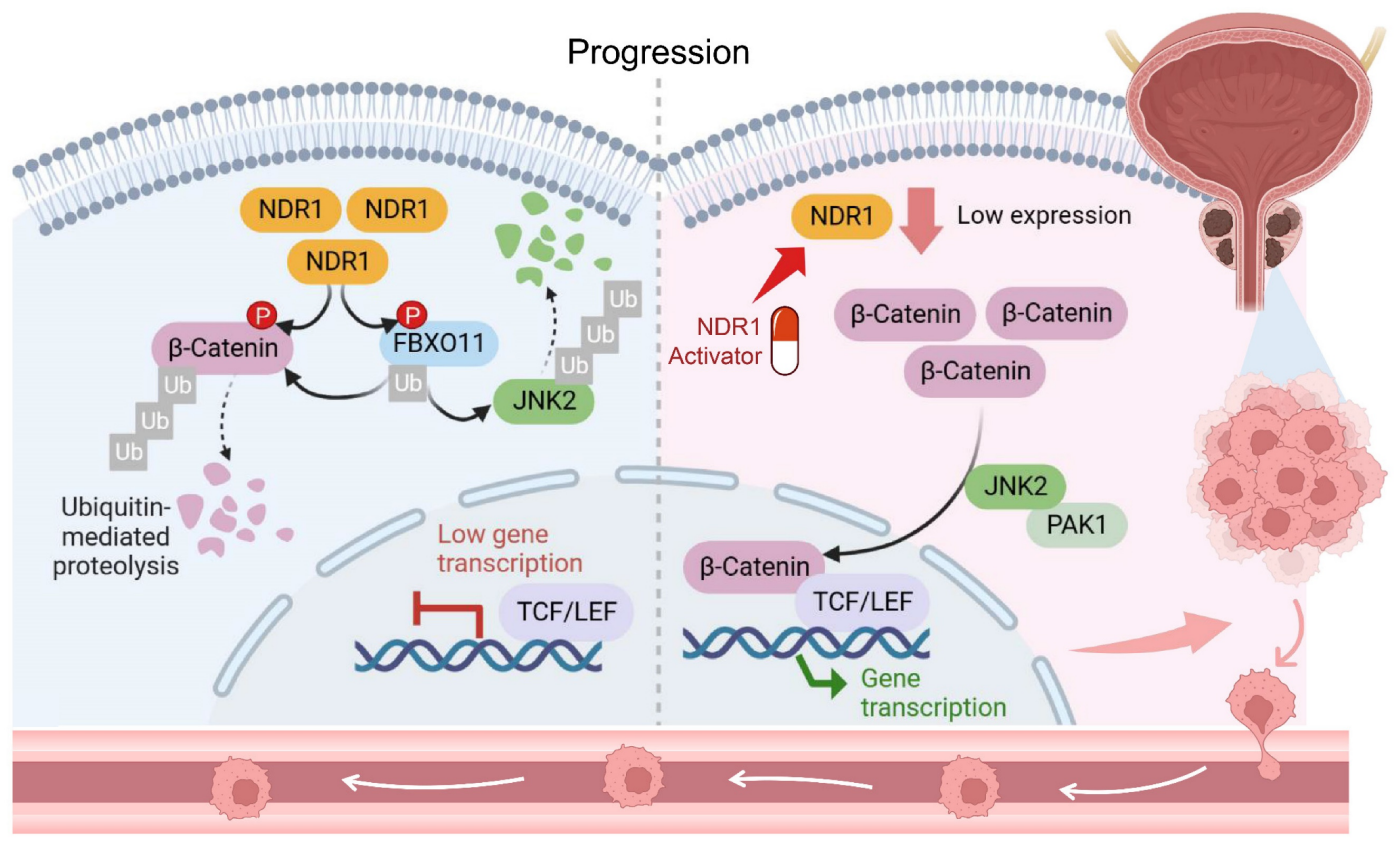
Schematic representation of the NDR1-FBXO11-β-catenin signaling axis and their significance in prostate cancer metastasis.

Taken together, a novel mechanism underlying dysregulated β-catenin expression in prostate cancer was identified in the study (**Fig. 8**). NDR1 mediates dual phosphorylation of both FBXO11 and β-catenin. On the one hand, NDR1 activates FBXO11, and on the other hand, it marks β-catenin for degradation by facilitating its binding with the E3 ubiquitin ligase FBXO11, which mediates β-catenin ubiquitination. Additionally, NDR1 phosphorylates FBXO11 to promote JNK2 ubiquitination and degradation, thereby controlling β-catenin nuclear translocation. Through these pathways, NDR1 maintains β-catenin transcriptional homeostasis. In prostate cancer cells with low NDR1 expression, the levels of β-catenin become dysregulated. Reduced levels of FBXO11 and rates of β-catenin phosphorylation lead to β-catenin ubiquitination suppression, resulting in the cytoplasmic accumulation of β-catenin. Simultaneously, elevated JNK2 expression leads to increased β-catenin nuclear translocation, where it acts as a transcription factor, promoting the expression of EMT genes and facilitating the EMT and metastasis of prostate cancer cells.

## 4. Discussion

### 4.1 Main interpretation

This study elucidates how NDR1 regulates β-catenin's transcriptional activity in prostate cancer through phosphorylation-dependent ubiquitination, promoting its degradation via FBXO11 binding. Additionally, the NDR1-FBXO11 axis regulates JNK2 ubiquitination, affecting β-catenin's nuclear translocation. Reduced NDR1 levels disrupt this regulatory mechanism, leading to increased β-catenin-mediated EMT and metastasis. Furthermore, aNDR1, a small-molecule agonist targeting NDR1, effectively inhibits prostate cancer metastasis, suggesting a promising therapeutic avenue.

Our investigations on the significance of NDR1 overexpression in prostate cancer reveal its inhibitory role in the EMT process. Clinical data indicates that lower NDR1 expression correlates with advanced disease stage and poorer prognosis in prostate cancer patients. Functional assays demonstrate that NDR1 overexpression reduces migratory and invasive capacities of prostate cancer cells by inhibiting EMT, which may not be related to the proliferation rate of the cells since the upper chamber had a lower serum concentration (0.2% BSA) than the upper chamber (20% FBS) and as evidenced by altered expression of epithelial and mesenchymal markers. These findings are concordant with those of Yue *et al.* 51, who reported that NDR1 silencing contributed to prostate cancer cell metastasis by activating EMT. Similarly, in the context of prostate cancer, Bai *et al.* 36 reported that NDR1 hindered the spread of prostate cancer cells by dampening EMT, with its reduced levels associated with worsened prognosis, highlighting the substantial promise of NDR1 in combating tumorigenesis. However, compared to their studies, our study delves deeper into the molecular mechanisms underlying this process, particularly focusing on the phosphorylation-mediated regulation of FBXO11 and β-catenin, and the downstream effects on EMT and metastasis, shedding light on specific molecular pathways involved in prostate cancer progression.

Understanding the NDR1-FBXO11-β-catenin axis provides a basis for developing targeted therapies that aim to modulate this pathway. Mechanistically, NDR1 kinase activity was found to play an essential role in inhibiting EMT, as shown by experiments with a kinase-deficient NDR1 variantMoreover, our results also showed that NDR1 promotes β-catenin ubiquitination, independent of its effects on proliferation, thus inhibiting EMT-related processes. Further investigations reveal that NDR1 phosphorylates FBXO11 at Ser187, enhancing its ubiquitination of β-catenin, which regulates EMT and prostate cancer progression. Additionally, NDR1 phosphorylates β-catenin at Ser33/37, promoting its ubiquitination and degradation. Through these mechanisms, the NDR1-FBXO11-β-catenin axis regulates prostate cancer metastasis, as confirmed *in vivo*. The clinical relevance of these findings could be as follows. First, NDR1 expression levels and its downstream effectors like FBXO11 and β-catenin could serve as prognostic markers for prostate cancer progression and metastasis since patients with lower NDR1 expression or dysregulated activity in this pathway may have a higher risk of aggressive disease and poor outcomes. Second, combining therapies targeting NDR1 with existing treatments like androgen deprivation therapy (ADT) or chemotherapy could enhance treatment efficacy and overcome resistance mechanisms 52-54. By inhibiting EMT and metastasis, such combination approaches may improve patient outcomes and survival rates. Third, stratifying patients based on the status of the NDR1-FBXO11-β-catenin pathway could facilitate personalized treatment strategies. Patients with alterations in this pathway may benefit more from therapies targeting NDR1 or its downstream effectors, leading to tailored treatment approaches.

Recently, Bai *et al.* 36 discovered a novel small-molecule agonist, named aNDR1, targeting NDR1 for prostate cancer therapy. Their compound specifically binds to NDR1, promoting its expression, enzymatic activity, and phosphorylation. In addition, aNDR1 exhibits favorable drug-like properties and selectively inhibits prostate cancer cell proliferation and migration while promoting apoptosis both *in vitro* and *in vivo*. Moreover, aNDR1 demonstrates efficacy in inhibiting subcutaneous tumors and lung metastatic nodules without apparent toxicity, highlighting the potential of aNDR1 as a lead compound for clinical therapy in prostate cancer. Compared to the study by Bai *et al.*, our study provides a more comprehensive understanding of the molecular mechanisms underlying the therapeutic potential of NDR1 in prostate cancer. While Bai *et al.* focused on the development of a small-molecule agonist targeting NDR1, our study delves deeper into the mechanistic insights behind NDR1's role in regulating β-catenin's transcriptional activity and its impact on prostate cancer progression. Moreover, by identifying the NDR1-FBXO11-β-catenin axis and its role in regulating EMT and metastasis, our findings provide a more detailed understanding of NDR1's antitumorigenic effects. Furthermore, our study demonstrates the potential clinical relevance of targeting NDR1 for prostate cancer therapy beyond the development of aNDR1. By uncovering the molecular mechanisms underlying NDR1's inhibitory effects on EMT and metastasis, we lay a firmer foundation for the development of novel therapeutic strategies targeting this pathway. Additionally, our findings suggest that NDR1 inhibition may lead to dysregulated β-catenin signaling, emphasizing the importance of NDR1 as a potential therapeutic target in prostate cancer.

The transcriptional coactivator β-catenin is regulated via multiple mechanisms, and the degradation of this protein is a key event in this signaling cascade. In quiescent cells, the cellular abundance of β-catenin remains low due to the activity of a multisubunit destruction complex comprising AXIN1, APC, CK1, and GSK3β. This complex sequesters and phosphorylates β-catenin, which is subsequently ubiquitinated by the E3 ligase β-TrCP, leading to its degradation 55-58. In addition to the classical β-catenin degradation pathway, several non-classical negative feedback regulators are involved in maintaining β-catenin stability. For example, AXIN2 has been reported to enhance cytoplasmic destruction complex activity and downregulate β-catenin-mediated transcription 59. RNF43 and ZNRF3 mediate WNT receptor ubiquitination, driving its internalization and lysosomal degradation, thereby reducing cell sensitivity to WNT signaling 60. Herein, we identified a non-canonical β-catenin ubiquitination pathway wherein NDR1 phosphorylates β-catenin at Ser33/37, facilitating FBXO11-mediated ubiquitination and degradation, distinct from the classical GSK3β-mediated phosphorylation and β-TrCP ubiquitination pathway. Additionally, this pathway involves JNK2 ubiquitination, further preventing β-catenin nuclear translocation and transcriptional activity. However, considering the complexity of β-catenin regulation, further research is needed to explore crosstalk among the NDR1-FBXO11-β-catenin signaling axis and other nonclassical pathways.

### 4.2 Limitations

Recent studies have revealed various biological functions of nuclear β-catenin transcriptional effects. The sustained abnormal activation of β-catenin in cancer cells has been associated with self-renewing growth properties 61. The β-catenin/TCF4 transcriptional complex directly binds to the GPX4 promoter region and induces its expression, thereby inhibiting ferroptosis in gastric cancer cells 62. GABA-mediated β-catenin activation stimulates tumor cell proliferation while inhibiting CD8 T-cell infiltration, leading to tumor immune evasion 63. Aberrant β-catenin activation is also associated with drug resistance in various anti-cancer therapies 64-68. This study primarily demonstrates the role of β-catenin in promoting EMT in prostate cancer, elucidating how it influences tumor metastasis behavior via post-translational modifications and transcription factor activity dysregulation. However, given the multifaceted nature of β-catenin's functionality, its abnormal activation in prostate cancer cells might also impact other tumor biological behaviors and, therefore, urges the need for further research to fully understand this possibility.

## 5. Conclusions

In summary, this study unveils the association between NDR1 and prostate cancer, shedding light on its regulatory mechanism over β-catenin via a phosphorylation-dependent ubiquitination pathway mediated by FBXO11. The discovery of this nonclassical ubiquitination degradation pathway for β-catenin, distinct from conventional pathways, enhances our comprehension of dysregulation mechanisms involving β-catenin. Furthermore, the therapeutic efficacy of an NDR1 small molecule in prostate cancer has been confirmed. These findings not only establish a new theoretical framework for comprehending the mechanisms underlying prostate cancer metastasis but also offer valuable insights for the future development of relevant therapeutic strategies and drugs, paving the way for clinical translation.

## Supplementary Material

Supplementary figures and tables.

## Abbreviations

EMT: Epithelial-Mesenchymal Transition
APC: Adenomatous Polyposis Coli
GSK-3β: Glycogen Synthase Kinase-3β
CK1: Casein Kinase 1
IP: Immunoprecipitation
RT-PCR: Reverse Transcription Polymerase Chain Reaction
CHX: cycloheximide
ANOVA: one-way analysis of variance
TGF-β: Transforming growth factor-beta
LC-MS/MS: Liquid Chromatography-Tandem Mass Spectrometry
NPCs: nuclear pore complexes
JNK2: c-Jun N-terminal kinase 2
PAK1: p21 activated kinase 1
HE: haemoxylin-eosin
ADT: androgen deprivation therapy
