# Supplementary material for: NDR1/FBXO11 promotes phosphorylation-mediated ubiquitination of β-catenin to suppress metastasis in prostate cancer
Source: Int J Biol Sci. 2024 Sep 16;20(12):4957–77. doi: 10.7150/ijbs.98907 (PMC11414387; doi:10.7150/ijbs.98907)
Supplement: Supplementary file 1 — Supplementary figures and tables. [file ijbsv20p4957s1.pdf]

Supplementary Figure S1. Experimental analysis of NDR1-mediated regulation of EMT and β-catenin pathway in prostate cancer cells. A, B: Migration and scratch assays demonstrate that NDR1 inhibits epithelial-mesenchymal transition (EMT) in prostate cancer cells (DU145) by reducing cell migration and wound closure. C: Mutation of the NDR1 ATP-binding site (K118A) significantly reduces the phosphorylation rate of NDR1. D: Efficiency verification of β-TrCP knockout, confirming successful elimination of β-TrCP protein. E-G: The gray value quantification for protein half-life experiments. H: Immunoprecipitation (IP) with exogenous proteins confirms the direct interaction between FBXO11 and β-Catenin. I: IP with exogenous proteins confirms the direct interaction between NDR1 and β-Catenin. J: IP with exogenous proteins confirms the direct interaction between NDR1 and FBXO11. K: Measurement of panphosphorylation levels of IP-enriched FBXO11 reveals significant promotion of FBXO11 phosphorylation by NDR1. L: NDR1 promotes phosphorylation of β-Catenin at residues S33/37. M: Changes in membrane expression of β-catenin after stimulation with TGF-β. N: Efficiency verification of FBXO11 knockout, confirming successful down-expression of the FBXO11 protein. O: IP binding assay demonstrates that mutation of FBXO11 at residue S187A affects its interaction with β-catenin.

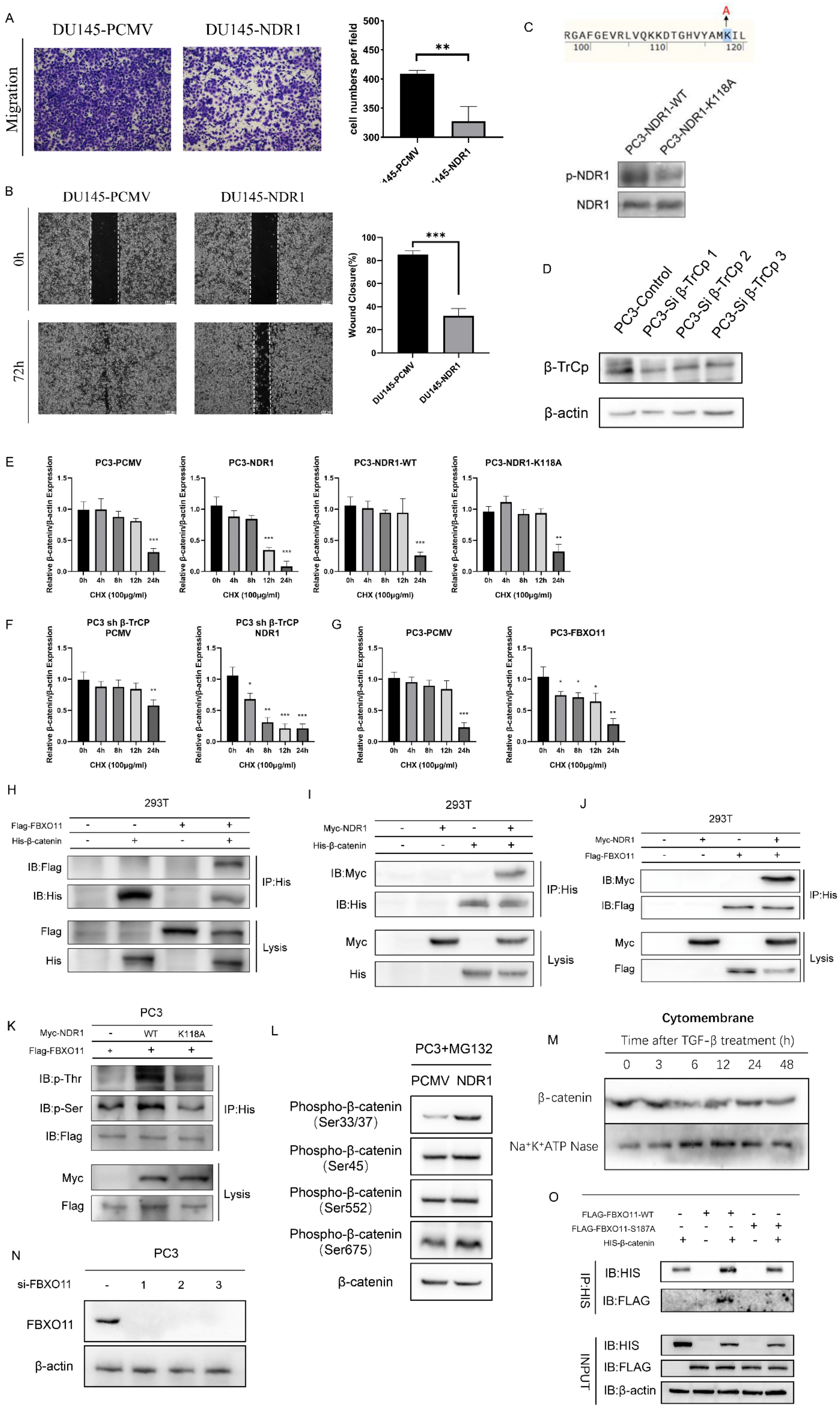

Supplementary Figure S2. Secondary mass spectrometry (MS/MS) spectrum of phosphorylated FBXO11 peptide. Control Spectrum of Phosphorylated FBXO11 Peptide at (A) Serine 140 and 143, (B) Serine 147, (C) Serine 542, and (D) Serine 841.

A FBXO11 Ser140 Ser143 :VSGKSQDLSAAPAEQYLQEK (NC-FBXO11-293T)

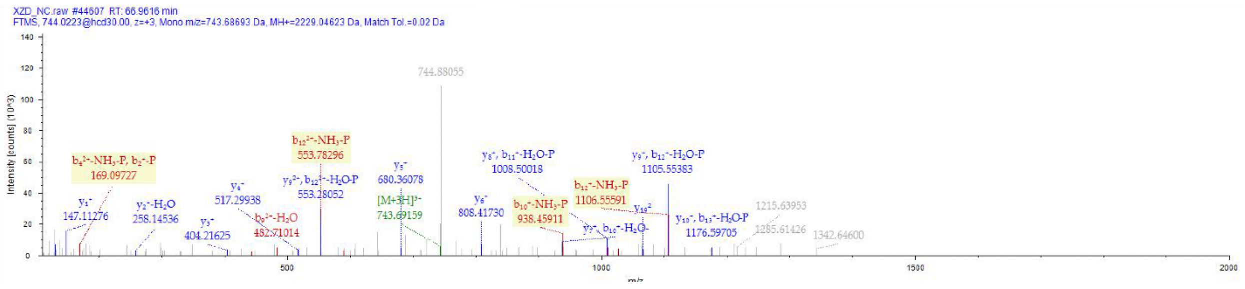

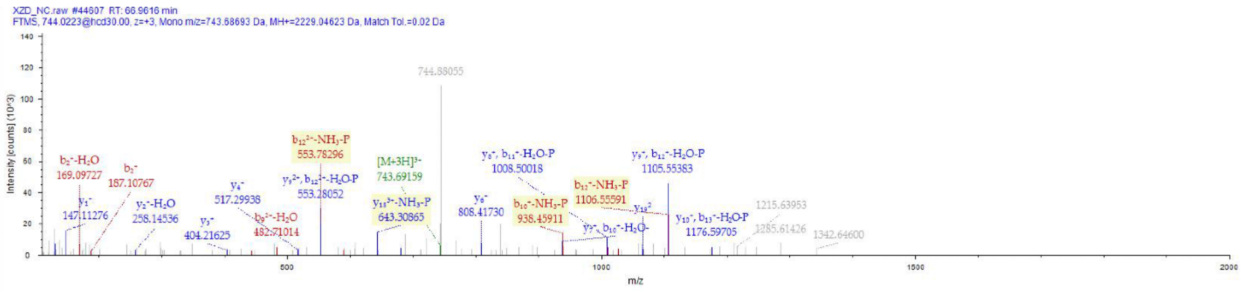

B FBXO11 Ser147:VSGKSQDLSAAPAEQYLQEK (NC-FBXO11-293T)

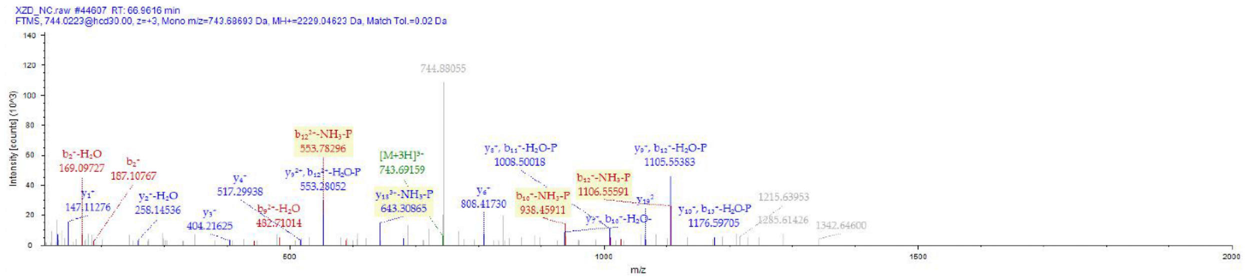

C FBXO11 Ser542: GNSIFNGNQGGVYIFGDGR (NC-FBXO11-293T)

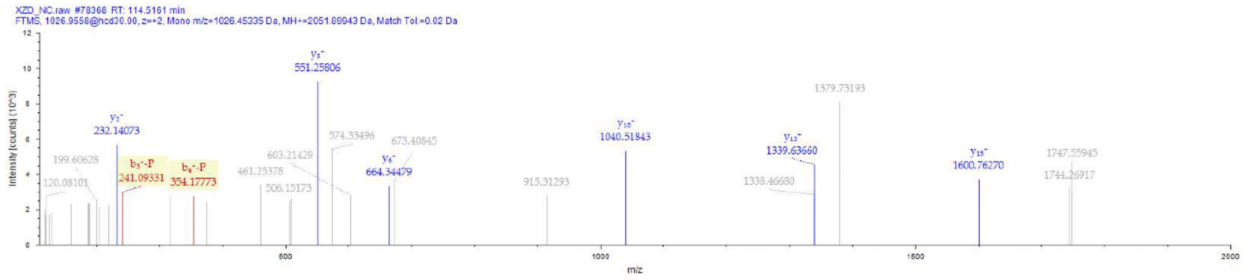

D FBXO11 Ser841: ISSYTSYPMHDFYR (NC-FBXO11-293T)

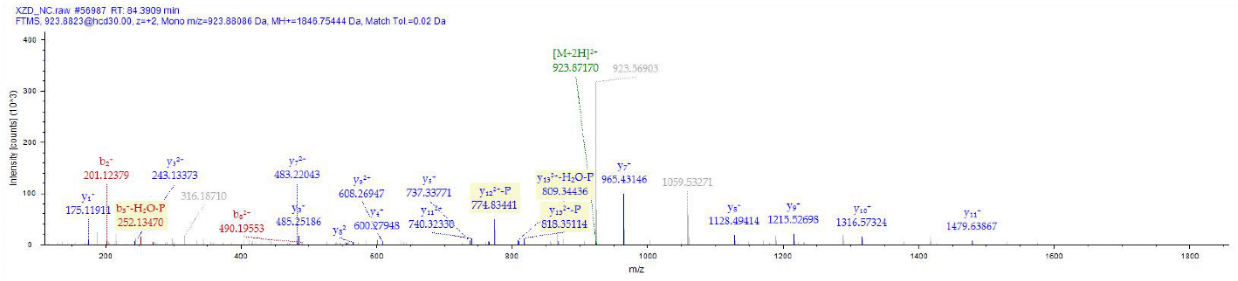

Supplementary Figure S3. Secondary mass spectrometry (MS/MS) spectrum of phosphorylated FBXO11 peptide with NDR1 co-expression. Spectrum of phosphorylated FBXO11 peptide at (A) Serine 140 and 143, (B) Serine 147, (C) Serine 542, and (D) Serine 841 with NDR1 co-expression.

A FBXO11 Ser140 Ser143 :VSGKSQDLSAAPAEQYLQEK (NDR1-FBXO11-293T)

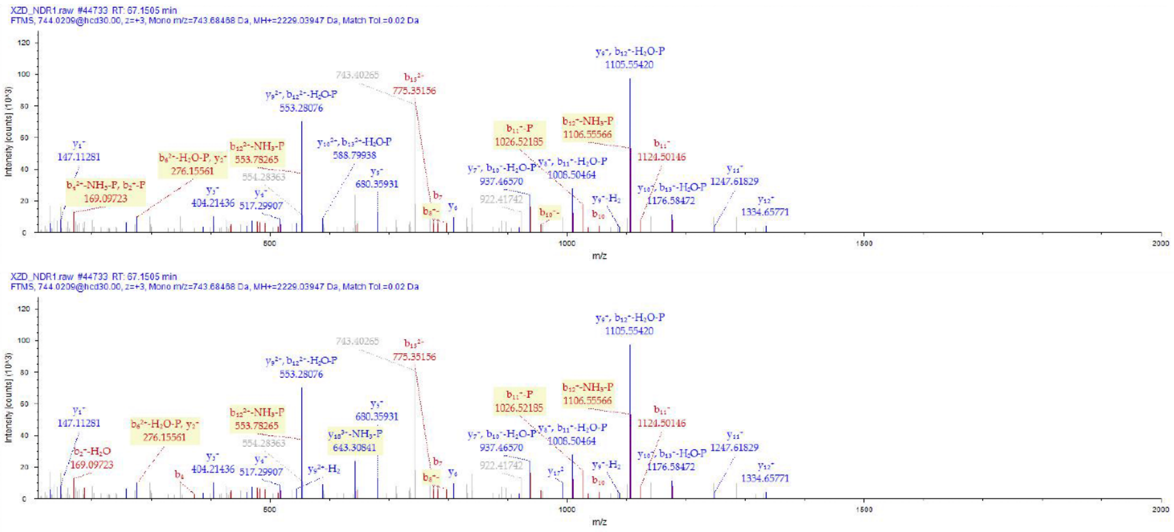

B FBXO11 Ser147:VSGKSQDLSAAPAEQYLQEK (NDR1-FBXO11-293T)

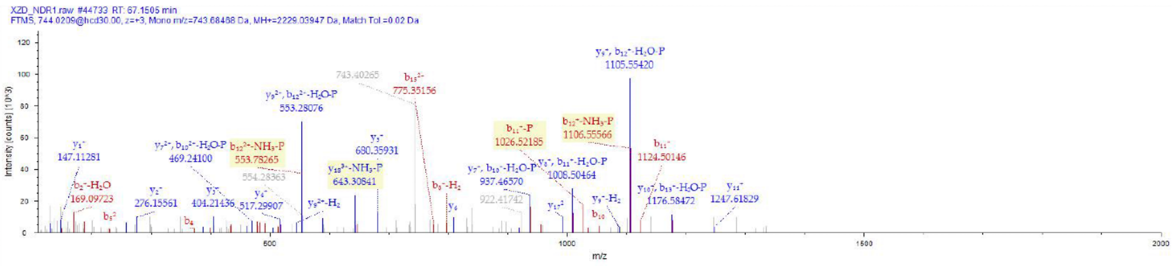

C FBXO11 Ser542: FSELANDPILWK (NDR1-FBXO11-293T)

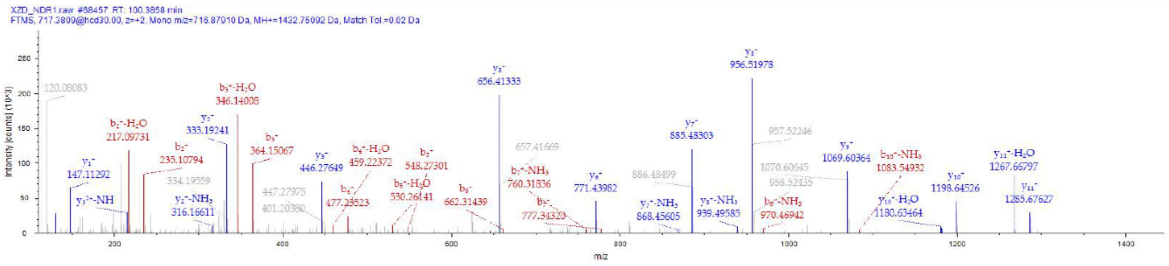

D NDR1 Ser281: QLAFSTVGTPDYIAPEVFMQTGYNK

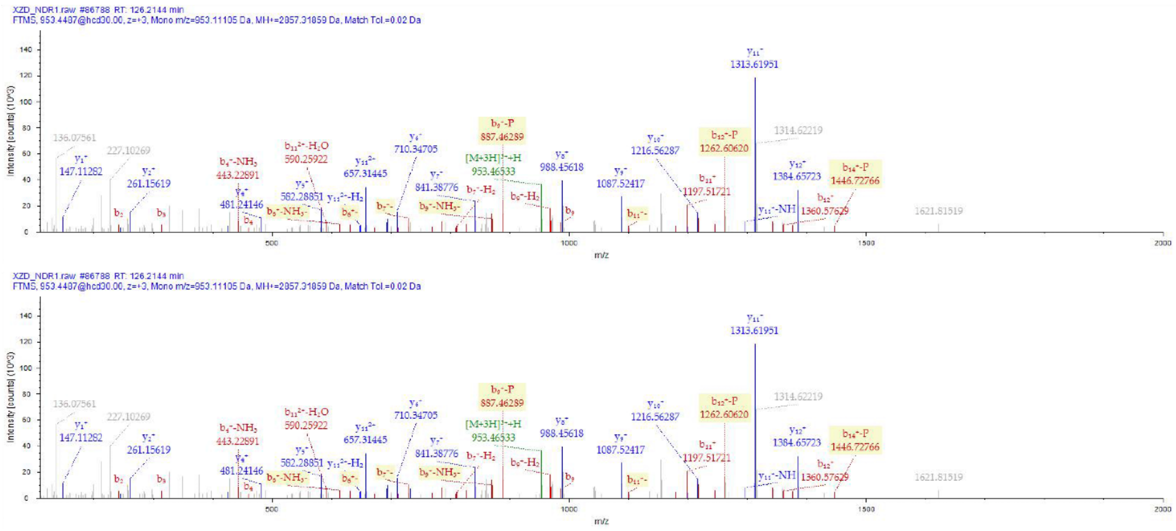

Supplementary Figure S4. Bioinformatics and experimental analyses of β-catenin and FBXO11 regulatory pathways. A: Predicted post-translational modification sites of β-catenin. B: Conservation analysis of FBXO11 protein sequence across different species. C: Screening of small molecule activators for NDR1, showing the process of identifying compounds that enhance NDR1 activity as potential therapeutic agents. D: Prediction of FBXO11-mediated ubiquitination of β-catenin and PAK1, indicating the computational modeling of the interaction between FBXO11 and its substrates for ubiquitination, β-catenin, and PAK1.

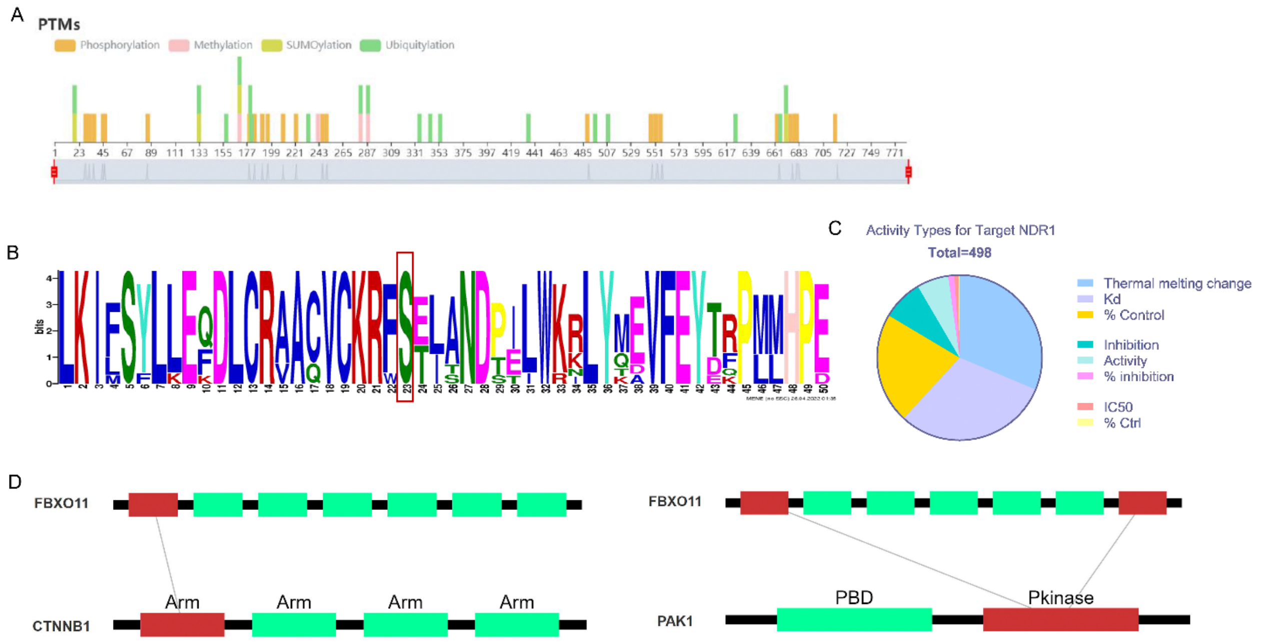

Supplementary Table S1. Sequences of primers used in RT-qPCR assay.

Gene Name		Primer sequences (5' to 3')
GAPDH	Forward primer	GAGTCAACGGATTTGGTCGT
	Reverse primer	GACAAGCTTCCCGTTCTCAG
STK38	Forward primer	TCAGCACATGCTCGGAAGG
	Reverse primer	ACAAGCCGTACCTCACCAAAT
CTNNB1	Forward primer	CTTGGACTGAGACTGCTGATCTTG
	Reverse primer	CACCAGAGTGAAAAGAACGATAGCTA
STK38-K118A	Forward primer	TGTATGCAATGGCTATACTCCGTAAAGCAGATATG
	Reverse primer	GAGTATAGCCATTGCATACACATGTCCCGTATCTT
FBXO11	Forward primer	TGGACGTGATGTTGGTGTGTTCACA
	Reverse primer	TCCTCCAGTCTGCCCATGGTGA
c-Myc	Forward primer	GGCTCCTGGCAAAAGGTCA
	Reverse primer	CTGCGTAGTTGTGCTGATGT
Cyclin D1	Forward primer	GCTGCGAAGTGGAAACCATC
	Reverse primer	CCTCCTTCTGCACACATTTGAA
CTNNB1	Forward primer	GCGGTACCATGGCTACTCAAGCT
	Reverse primer	CGGCCGCTCTAGACTCGAGTTTA
CTNNB1 S33A	Forward primer	CAGCAACAGTCTTACCTGGACGCTGGAATCCATTCT
	Reverse primer	AGTGGCACCAGAATGGATTCCAGCGTCCAGGTAAGA
CTNNB1 S37A	Forward primer	TACCTGGACTCTGGAATCCATGCTGGTGCCACTACC
	Reverse primer	AGGAGCTGTGGTAGTGGCACCAGCATGGATTCCAGA

CTNNB1 S33A-S37A	Forward primer	TACCTGGACGCTGGAATCCATGCTGGTGCCACTACC
	Reverse primer	AGGAGCTGTGGTAGTGGCACCAGCATGGATTCCAGC
CTNNB1 S1	Forward primer	GCGTCGACAAAAAGCGGCTGTT
	Reverse primer	GCGTCTAGATTATGTGGCAAGTTCTGC
CTNNB1 S2	Forward primer	CGTCGACAACGTGCAATCCCTGAA
	Reverse primer	CGCGCGTCTAGATTAACTAGCCAGTATG
CTNNB1 S3	Forward primer	CGTCGACAAGGTGGACCCCAAG
	Reverse primer	CGCGCGTCTAGATTATAGTCCTCTGATAAC
CTNNB1 S4	Forward primer	GCGGCGTCGACAAAATACCATTCCATT
	Reverse primer	GGCGCGTCTAGATTACAGGTCAGTATCA

Supplementary Table S2. Correlation of NDR1 expression with prostate cancer characteristics.

Characteristics	Low expression of NDR1	High expression of NDR1	p value
n	249	250	
T stage，n (%)			0.023
T2	109 (22.2%)	80 (16.3%)	
T3	133 (27%)	159 (32.3%)	
T4	4 (0.8%)	7 (1.4%)	
N stage，n (%)			0.047
N0	173 (40.6%)	174 (40.8%)	
N1	29 (6.8%)	50 (11.7%)	
M stage，n (%)			1.000
M0	221 (48.3%)	234 (51.1%)	
M1	1 (0.2%)	2 (0.4%)	
PSA (ng/ml)，n (%)			0.136
＜4	208 (47.1%)	207 (46.8%)	
≥4	9 (2%)	18 (4.1%)	
Gleason score，n (%)			0.012
6	27 (5.4%)	19 (3.8%)	
7	132 (26.5%)	115 (23%)	
8	35 (7%)	29 (5.8%)	
9	55 (11%)	83 (16.6%)	
10	0 (0%)	4 (0.8%)	

Supplementary Table 3. NetPhos3.1 predicts phosphorylation sites on FBXO11 by NDR1 (Score > 0.5).

>AAV87312.1 927 amino acids
#
netphos-3.1b prediction results
#

# Sequence	# x	Context	Score	Kinase	Answer
# AAV87312.1	3 S	--MNSVRAA	0.799	PKC	YES
#					
# AAV87312.1	15 S	PRRVSRPRP	0.998	unsp	YES
# AAV87312.1	15 S	PRRVSRPRP	0.834	PKB	YES
# AAV87312.1	15 S	PRRVSRPRP	0.788	PKA	YES
# AAV87312.1	15 S	PRRVSRPRP	0.530	PKC	YES
# AAV87312.1	15 S	PRRVSRPRP	0.510	RSK	YES
#					
# AAV87312.1	90 S	VAEESGPGA	0.573	CKI	YES
#					
# AAV87312.1	97 S	GAQNSPYQL	0.837	unsp	YES
# AAV87312.1	97 S	GAQNSPYQL	0.583	cdk5	YES
# AAV87312.1	97 S	GAQNSPYQL	0.510	p38MAPK	YES
#					
# AAV87312.1	105 T	LRRKTLLPK	0.895	unsp	YES
# AAV87312.1	105 T	LRRKTLLPK	0.769	PKA	YES
# AAV87312.1	105 T	LRRKTLLPK	0.664	PKG	YES
#					
# AAV87312.1	111 T	LPKRTACPT	0.670	PKG	YES
# AAV87312.1	111 T	LPKRTACPT	0.536	unsp	YES
#					
# AAV87312.1	115 T	TACPTKNSM	0.608	PKC	YES
#					
# AAV87312.1	118 S	PTKNSMEGA	0.758	unsp	YES
# AAV87312.1	118 S	PTKNSMEGA	0.577	cdc2	YES
#					
# AAV87312.1	123 S	MEGASTSTT	0.954	unsp	YES
#					
# AAV87312.1	124 T	EGASTSTTE	0.548	CKII	YES
#					
# AAV87312.1	125 S	GASTSTTEN	0.977	unsp	YES
# AAV87312.1	125 S	GASTSTTEN	0.537	CKI	YES
# AAV87312.1	125 S	GASTSTTEN	0.510	CKII	YES
#					
# AAV87312.1	140 S	RARVSGKSQ	0.998	unsp	YES
# AAV87312.1	140 S	RARVSGKSQ	0.849	PKC	YES
# AAV87312.1	140 S	RARVSGKSQ	0.738	PKA	YES
#					
# AAV87312.1	143 S	VSGKSQDLS	0.915	unsp	YES
#					
# AAV87312.1	147 S	SQDLSAAPA	0.626	unsp	YES
#					
# AAV87312.1	154 Y	PAEQYLQEK	0.667	unsp	YES
#					
# AAV87312.1	187 S	CKRFSELAN	0.956	unsp	YES
# AAV87312.1	187 S	CKRFSELAN	0.565	PKA	YES
#					
# AAV87312.1	200 Y	WKRLYMEVF	0.520	EGFR	YES
#					
# AAV87312.1	206 Y	EVFEYTRPM	0.502	unsp	YES
#					
# AAV87312.1	226 Y	NPEEYEHPN	0.962	unsp	YES
# AAV87312.1	226 Y	NPEEYEHPN	0.553	EGFR	YES
#					
# AAV87312.1	254 Y	AEHFYSNPA	0.950	unsp	YES
# AAV87312.1	254 Y	AEHFYSNPA	0.536	EGFR	YES
#					
# AAV87312.1	269 Y	NMLYYDTIE	0.566	unsp	YES

```
#
# AAV87312.1    271 T   LYYDTIEDA   0.708   unsp      YES
#
# AAV87312.1    295 Y   HSGIYTDEW   0.546   unsp      YES
#
# AAV87312.1    296 T   SGIYTDEWI   0.582   CKII      YES
#
# AAV87312.1    301 Y   DEWIYIESP   0.973   unsp      YES
# AAV87312.1    301 Y   DEWIYIESP   0.531   EGFR      YES
# AAV87312.1    301 Y   DEWIYIESP   0.519   SRC       YES
#
# AAV87312.1    325 T   IIENTRDST   0.792   unsp      YES
#
# AAV87312.1    328 S   NTRDSTFVF   0.902   unsp      YES
# AAV87312.1    328 S   NTRDSTFVF   0.573   PKA       YES
#
# AAV87312.1    336 S   FMEGSEDAY   0.526   CKII      YES
#
# AAV87312.1    340 Y   SEDAYVGYM   0.984   unsp      YES
#
# AAV87312.1    345 T   VGYMTIRFN   0.717   PKC       YES
# AAV87312.1    345 T   VGYMTIRFN   0.601   unsp      YES
#
# AAV87312.1    382 T   IIRSTCTVG   0.558   PKC       YES
#
# AAV87312.1    384 T   RSTCTVGSA   0.783   PKC       YES
#
# AAV87312.1    399 T   GACPTIKHC   0.681   PKC       YES
#
# AAV87312.1    406 S   HCNISDCEN   0.937   unsp      YES
# AAV87312.1    406 S   HCNISDCEN   0.562   CKII      YES
# AAV87312.1    406 S   HCNISDCEN   0.558   PKA       YES
#
# AAV87312.1    423 Y   AQGIYEDNE   0.931   unsp      YES
#
# AAV87312.1    487 Y   EVKAYANPT   0.808   unsp      YES
#
# AAV87312.1    491 T   YANPTVVRC   0.626   PKC       YES
#
# AAV87312.1    521 Y   ENKIYANNF   0.929   unsp      YES
# AAV87312.1    521 Y   ENKIYANNF   0.518   INSR      YES
#
# AAV87312.1    532 S   VWITSNSDP   0.813   unsp      YES
#
# AAV87312.1    537 T   NSDPTIRGN   0.765   unsp      YES
# AAV87312.1    537 T   NSDPTIRGN   0.672   PKC       YES
#
# AAV87312.1    542 S   IRGNSIFNG   0.893   unsp      YES
# AAV87312.1    542 S   IRGNSIFNG   0.777   PKA       YES
#
# AAV87312.1    567 Y   GNDIYGNAL   0.939   unsp      YES
# AAV87312.1    567 Y   GNDIYGNAL   0.587   INSR      YES
# AAV87312.1    567 Y   GNDIYGNAL   0.548   EGFR      YES
#
# AAV87312.1    580 S   IRTNSCPIV   0.732   PKA       YES
#
# AAV87312.1    613 Y   ENEVYSNTL   0.974   unsp      YES
# AAV87312.1    613 Y   ENEVYSNTL   0.518   INSR      YES
#
# AAV87312.1    616 T   VYSNTLAGV   0.533   CKI       YES
#
# AAV87312.1    623 T   GVWVTTGST   0.747   PKC       YES
#
# AAV87312.1    627 T   TTGSTPVLR   0.647   cdk5      YES
# AAV87312.1    627 T   TTGSTPVLR   0.536   p38MAPK   YES
#
```

# AAV87312.1	637 S	NRIHSGKQV	0.994	unsp	YES
# AAV87312.1	637 S	NRIHSGKQV	0.848	PKC	YES
# AAV87312.1	637 S	NRIHSGKQV	0.626	PKA	YES
# AAV87312.1	637 S	NRIHSGKQV	0.600	PKB	YES
#					
# AAV87312.1	659 Y	DNDIYNHMY	0.953	unsp	YES
# AAV87312.1	659 Y	DNDIYNHMY	0.560	EGFR	YES
# AAV87312.1	659 Y	DNDIYNHMY	0.554	INSR	YES
# AAV87312.1	659 Y	DNDIYNHMY	0.536	SRC	YES
#					
# AAV87312.1	672 S	IRTGSNPKI	0.597	PKC	YES
# AAV87312.1	672 S	IRTGSNPKI	0.567	RSK	YES
#					
# AAV87312.1	721 T	DSNPTLRRN	0.902	PKC	YES
# AAV87312.1	721 T	DSNPTLRRN	0.570	unsp	YES
#					
# AAV87312.1	761 S	GVLISTNSH	0.673	PKC	YES
#					
# AAV87312.1	790 T	HATATLEGN	0.822	PKC	YES
#					
# AAV87312.1	813 T	GVNVTMKDN	0.853	PKC	YES
#					
# AAV87312.1	831 S	EKAVSRGQC	0.787	unsp	YES
#					
# AAV87312.1	840 S	LYKISSYTS	0.556	PKG	YES
#					
# AAV87312.1	841 S	YKISSYTSY	0.764	PKC	YES
# AAV87312.1	841 S	YKISSYTSY	0.548	DNAPK	YES
#					
# AAV87312.1	844 S	SSYTSYPMH	0.990	unsp	YES
#					
# AAV87312.1	845 Y	SYTSYPMHD	0.506	unsp	YES
#					
# AAV87312.1	858 T	HTCNTTDRN	0.715	PKC	YES
# AAV87312.1	858 T	HTCNTTDRN	0.519	unsp	YES
#					
# AAV87312.1	896 S	AGTLSNPCT	0.569	cdc2	YES
# AAV87312.1	896 S	AGTLSNPCT	0.509	PKA	YES
#					
# AAV87312.1	900 T	SNPCTLASE	0.512	CKI	YES
#					
# AAV87312.1	903 S	CTLASEPTH	0.510	cdc2	YES
#					
# AAV87312.1	906 T	ASEPTHDTD	0.553	CKI	YES
#					
# AAV87312.1	911 T	HDTDTLYDS	0.866	unsp	YES
#					
# AAV87312.1	913 Y	TDTLYDSAP	0.941	unsp	YES
# AAV87312.1	913 Y	TDTLYDSAP	0.561	SRC	YES
#					
# AAV87312.1	915 S	TLYDSAPPI	0.517	cdc2	YES

MNSVRAANRRPRRVSRPRPVQQQQQQPPQQPPPQPPQQQPPQQQPPPPPQ	#	50
QQQQQQPPPPPPPPPPLPQERNNVGERDDDVPADMVAEESGPGAQNSPYQ	#	100
LRRKTLLPKRTACPTKNSMEGASTSTTENFGHRAKRARVSGKSQDLSAAP	#	150
AEQYLQEKLPDEVVLKIFSYLLEQDLCRAACVCKRFSELANDPILWKRLY	#	200
MEVFEYTRPMMHPEPGKFYQINPEEYEHPNPWKESFQQLYKGAHVKPGFA	#	250
EHFYSNPARYKGRENMLYYDTIEDALGGVQEAHFDGLIFVHSGIYTDEWI	#	300
YIESPITMIGAAPGKVADKVIIENTRDSTFVFMEGSEDAYVGYMTIRFNP	#	350
DDKSAQHHNAHHCLEITVNCSPIIDHCIIRSTCTVGSAVCVSGQGACPTI	#	400
KHCNISDCENVGLYITDHAQGIYEDNEISNNALAGIWVKNHGNPIIRRNH	#	450
IHHGRDVGVFTFDHGMGYFESCNIHRNRIAGFEVKAYANPTVVRCEIHHG	#	500
QTGGIYVHEKGRGQFIENKIYANNFAGVWITSNSDPTIRGNSIFNGNQGG	#	550
VYIFGDGRGLIEGNDIYGNALAGIQIRTNSCPIVRHNKIHDGQHGGIYVH	#	600
EKGQGVIEENEVYSNTLAGVWVTTGSTPVLRRNRIHSGKQVGVYFYDNGH	#	650
GVLEDNDIYNHMYSGVQIRTGSNPKIRRNKIWGGQNGGILVYNSGLGCIE	#	700

DNEIFDNAMAGVWIKTDSNPTLRRNKIHDGRDGGICIFNGGRGLLEENDI	#	750
FRNAQAGVLISTNSHPILRKNRIFDGFAAGIEITNHATATLEGNQIFNNR	#	800
FGGLFLASGVNVTMKDNKIMNNQDAIEKAVSRGQCLYKISSYTSYPMHDF	#	850
YRCHTCNTTDRNAICVNCIKKCHQGHDVEFIRHDRFFCDCGAGTLSNPCT	#	900
LASEPTHDTDTLYDSAPPIESNTLQHN	#	950

%1	..S...........S..................................	#	50
%1	....S......S...	#	100
%1	....T.....T...T..S....STS..............S..S...S...	#	150
%1	...Y...............................S...........Y	#	200
%1	.....Y..................Y........................	#	250
%1	...Y.............Y.T.......................YT....	#	300
%1	Y.......................T..S.......S...Y....T.....	#	350
%1	...............................T.T.............T.	#	400
%1	.....S...............Y...........................	#	450
%1	....................................Y...T.........	#	500
%1	....................Y.........S....T....S........	#	550
%1	................Y...........S....................	#	600
%1	............Y..T......T...T.........S.............	#	650
%1	........Y...........S............................	#	700
%1	....................T............................	#	750
%1	..........S............................T..........	#	800
%1	............T.................S........SS..SY.....	#	850
%1	.......T.....................................S...T	#	900
%1	..S..T....T.Y.S............		

Supplementary Table 4. Mass spectrometry results (phosphorylation sites).

NC-FBXO11-293T					
Position	Target	Modification	Classification	Highest Peptide Confidence	Sequence Motif
140	S	Phospho	Post-translational	High	AKRARVsGKSQDL
143	S	Phospho	Post-translational	High	ARVSGKsQDLSAA
147	S	Phospho	Post-translational	High	GKSQDLsAAPAEQ
542	S	Phospho	Post-translational	High	PTIRGNsIFNGNQ
841	S	Phospho	Post-translational	High	CLYKISsYTSYPM
NDR1-FBXO11-293T					
Position	Target	Modification	Classification	Highest Peptide Confidence	Sequence Motif
140	S	Phospho	Post-translational	High	AKRARVsGKSQDL
143	S	Phospho	Post-translational	High	ARVSGKsQDLSAA
147	S	Phospho	Post-translational	High	GKSQDLsAAPAEQ
187	S	Phospho	Post-translational	High	CVCKRFsELANDP

Supplementary Table 5. NetPhos3.1 predicts phosphorylation sites on β-catenin by NDR1 (Score > 0.5).

>CAA61107.1 781 amino acids
#
netphos-3.1b prediction results
#

# Sequence	# x	Context	Score	Kinase	Answer
# CAA61107.1	3 T	--MATQADL	0.565	DNAPK	YES
#					
# CAA61107.1	23 S	KAAVSHWQQ	0.749	PKC	YES
# CAA61107.1	23 S	KAAVSHWQQ	0.654	unsp	YES
#					
# CAA61107.1	30 Y	QQQSYLDSG	0.815	unsp	YES
#					
# CAA61107.1	33 S	SYLDSGIHS	0.564	PKC	YES
#					
# CAA61107.1	37 S	SGIHSGATT	0.525	PKA	YES
#					
# CAA61107.1	40 T	HSGATTTAP	0.806	PKC	YES
#					
# CAA61107.1	41 T	SGATTTAPS	0.766	unsp	YES
# CAA61107.1	41 T	SGATTTAPS	0.507	PKC	YES
#					
# CAA61107.1	45 S	TTAPSLSGK	0.838	unsp	YES
# CAA61107.1	45 S	TTAPSLSGK	0.600	CKI	YES
# CAA61107.1	45 S	TTAPSLSGK	0.572	PKC	YES
#					
# CAA61107.1	47 S	APSLSGKGN	0.995	unsp	YES
# CAA61107.1	47 S	APSLSGKGN	0.664	PKC	YES
# CAA61107.1	47 S	APSLSGKGN	0.605	CKI	YES
#					
# CAA61107.1	59 T	EDVDTSQVL	0.542	CKII	YES
#					
# CAA61107.1	60 S	DVDTSQVLY	0.665	ATM	YES
# CAA61107.1	60 S	DVDTSQVLY	0.597	DNAPK	YES
# CAA61107.1	60 S	DVDTSQVLY	0.512	CKII	YES
#					
# CAA61107.1	71 S	EQGFSQSFT	0.561	DNAPK	YES
# CAA61107.1	71 S	EQGFSQSFT	0.557	ATM	YES
# CAA61107.1	71 S	EQGFSQSFT	0.525	cdc2	YES
#					
# CAA61107.1	73 S	GFSQSFTQE	0.504	cdc2	YES
# CAA61107.1	73 S	GFSQSFTQE	0.500	PKC	YES
#					
# CAA61107.1	75 T	SQSFTQEQV	0.556	DNAPK	YES
#					
# CAA61107.1	86 Y	IDGQYAMTR	0.540	unsp	YES
#					
# CAA61107.1	89 T	QYAMTRAQR	0.589	PKC	YES
#					
# CAA61107.1	102 T	MFPETLDEG	0.694	unsp	YES
# CAA61107.1	102 T	MFPETLDEG	0.558	CKII	YES
#					
# CAA61107.1	111 S	MQIPSTQFD	0.572	cdc2	YES
#					
# CAA61107.1	112 T	QIPSTQFDA	0.511	ATM	YES
#					
# CAA61107.1	129 S	LAEPSQMLK	0.589	DNAPK	YES
#					
# CAA61107.1	179 S	VHQLSKKEA	0.997	unsp	YES
# CAA61107.1	179 S	VHQLSKKEA	0.780	PKC	YES
#					
# CAA61107.1	184 S	KKEASRHAI	0.848	unsp	YES
#					
# CAA61107.1	191 S	AIMRSPQMV	0.629	cdk5	YES
#					

# CAA61107.1	205 T	TMQNTNDVE	0.515	CKII	YES
#					
# CAA61107.1	210 T	NDVETARCT	0.768	unsp	YES
#					
# CAA61107.1	217 T	CTAGTLHNL	0.554	PKC	YES
#					
# CAA61107.1	222 S	LHNLSHHRE	0.937	unsp	YES
#					
# CAA61107.1	234 S	AIFKSGGIP	0.537	PKA	YES
#					
# CAA61107.1	246 S	KMLGSPVDS	0.979	unsp	YES
# CAA61107.1	246 S	KMLGSPVDS	0.534	p38MAPK	YES
# CAA61107.1	246 S	KMLGSPVDS	0.514	GSK3	YES
#					
# CAA61107.1	258 T	YAITTLHNL	0.717	unsp	YES
#					
# CAA61107.1	298 T	LAITTDCLQ	0.536	CKII	YES
#					
# CAA61107.1	311 S	GNQESKLII	0.537	cdc2	YES
#					
# CAA61107.1	330 T	NIMRTYTYE	0.636	PKC	YES
#					
# CAA61107.1	339 T	KLLWTTSRV	0.558	PKC	YES
# CAA61107.1	339 T	KLLWTTSRV	0.500	cdc2	YES
#					
# CAA61107.1	340 T	LLWTTSRVL	0.870	unsp	YES
# CAA61107.1	340 T	LLWTTSRVL	0.638	PKC	YES
#					
# CAA61107.1	352 S	SVCSSNKPA	0.750	PKC	YES
#					
# CAA61107.1	374 S	LTDPSQRLV	0.615	DNAPK	YES
# CAA61107.1	374 S	LTDPSQRLV	0.507	ATM	YES
#					
# CAA61107.1	384 T	NCLWTLRNL	0.545	PKC	YES
#					
# CAA61107.1	389 S	LRNLSDAAT	0.988	unsp	YES
# CAA61107.1	389 S	LRNLSDAAT	0.708	PKA	YES
#					
# CAA61107.1	393 T	SDAATKQEG	0.546	CKII	YES
#					
# CAA61107.1	411 S	QLLGSDDIN	0.522	cdc2	YES
#					
# CAA61107.1	432 Y	TCNNYKNKM	0.549	unsp	YES
#					
# CAA61107.1	472 T	LRHLTSRHQ	0.946	unsp	YES
#					
# CAA61107.1	547 T	AHQDTQRRT	0.973	unsp	YES
#					
# CAA61107.1	552 S	QRRTSMGGT	0.998	unsp	YES
# CAA61107.1	552 S	QRRTSMGGT	0.714	PKA	YES
#					
# CAA61107.1	556 T	SMGGTQQQF	0.567	PKC	YES
# CAA61107.1	556 T	SMGGTQQQF	0.549	DNAPK	YES
#					
# CAA61107.1	605 S	QLLYSPIEN	0.957	unsp	YES
# CAA61107.1	605 S	QLLYSPIEN	0.525	p38MAPK	YES
#					
# CAA61107.1	646 S	ELLHSRNEG	0.991	unsp	YES
#					
# CAA61107.1	653 T	EGVATYAAA	0.502	PKC	YES
#					
# CAA61107.1	663 S	LFRMSEDKP	0.718	unsp	YES
#					
# CAA61107.1	670 Y	KPQDYKKRL	0.527	INSR	YES
#					
# CAA61107.1	675 S	KKRLSVELT	0.997	unsp	YES

```
# CAA61107.1         675 S    KKRLSVELT    0.718    PKA     YES
#
# CAA61107.1         681 S    ELTSSLFRT    0.514    cdc2    YES
#
# CAA61107.1         715 S    QDDPSYRSF    0.992    unsp    YES
# CAA61107.1         715 S    QDDPSYRSF    0.874    PKC     YES
#
# CAA61107.1         716 Y    DDPSYRSFH    0.904    unsp    YES
# CAA61107.1         716 Y    DDPSYRSFH    0.522    INSR    YES
#
# CAA61107.1         718 S    PSYRSFHSG    0.642    unsp    YES
#
# CAA61107.1         724 Y    HSGGYGQDA    0.504    INSR    YES
```

```
MATQADLMELDMAMEPDRKAAVSHWQQQSYLDSGIHSGATTTAPSLSGKG     #     50
NPEEEDVDTSQVLYEWEQGFSQSFTQEQVADIDGQYAMTRAQRVRAAMFP     #     100
ETLDEGMQIPSTQFDAAHPTNVQRLAEPSQMLKHAVVNLINYQDDAELAT     #     150
RAIPELTKLLNDEDQVVVNKAAVMVHQLSKKEASRHAIMRSPQMVSAIVR     #     200
TMQNTNDVETARCTAGTLHNLSHHREGLLAIFKSGGIPALVKMLGSPVDS     #     250
VLFYAITTLHNLLLHQEGAKMAVRLAGGLQKMVALLNKTNVKFLAITTDC     #     300
LQILAYGNQESKLIILASGGPQALVNIMRTYTYEKLLWTTSRVLKVLSVC     #     350
SSNKPAIVEAGGMQALGLHLTDPSQRLVQNCLWTLRNLSDAATKQEGMEG     #     400
LLGTLVQLLGSDDINVVTCAAGILSNLTCNNYKNKMMVCQVGGIEALVRT     #     450
VLRAGDREDITEPAICALRHLTSRHQEAEMAQNAVRLHYGLPVVVKLLHP     #     500
PSHWPLIKATVGLIRNLALCPANHAPLREQGAIPRLVQLLVRAHQDTQRR     #     550
TSMGGTQQQFVEGVRMEEIVEGCTGALHILARDVHNRIVIRGLNTIPLFV     #     600
QLLYSPIENIQRVAAGVLCELAQDKEAAEAIEAEGATAPLTELLHSRNEG     #     650
VATYAAAVLFRMSEDKPQDYKKRLSVELTSSLFRTEPMAWNETADLGLDI     #     700
GAQGEPLGYRQDDPSYRSFHSGGYGQDALGMDPMMEHEMGGHHPGADYPV     #     750
DGLPDLGHAQDLMDGLPPGDSNQLAWFDTDL                        #     800
```

```
%1   ..T...................S......Y..S...S..TT...S.S...     #     50
%1   ........TS..........S.S.T..........Y..T...........     #     100
%1   .T........ST................S.....................     #     150
%1   ............................S...S......S.........     #     200
%1   ....T....T......T....S...........S...........S....     #     250
%1   .......T.......................................T..     #     300
%1   ..........S..................T........TT..........     #     350
%1   .S.....................S.........T....S...T.......     #     400
%1   ..........S....................Y..................     #     450
%1   .....................T............................     #     500
%1   ..............................................T...     #     550
%1   .S...T............................................     #     600
%1   ....S........................................S....     #     650
%1   ..T.........S......Y....S.....S...................     #     700
%1   ..............SY.S.....Y..........................     #     750
%1   ...............................
```
